# Supplementary material for: Pilot randomized trial of the effect of antibacterial mouthwash on muscle contractile function in healthy young adults
Source: PLoS One. 2025 Feb 12;20(2):e0312961. doi: 10.1371/journal.pone.0312961 (PMC11819566; doi:10.1371/journal.pone.0312961)
Supplement: S1 Data — (DOCX) [file pone.0312961.s001.docx]

| Table Analyzed | nitrate |  |  |  |  |
| --- | --- | --- | --- | --- | --- |
|  |  |  |  |  |  |
| Two-way RM ANOVA | Matching: Across row |  |  |  |  |
| Assume sphericity? | Yes |  |  |  |  |
| Alpha | 0.05 |  |  |  |  |
|  |  |  |  |  |  |
| Source of Variation | % of total variation | P value | P value summary | Significant? |  |
| Group x Time | 3.318 | 0.0765 | ns | No |  |
| Group | 0.7009 | 0.6329 | ns | No |  |
| Time | 0.8719 | 0.3520 | ns | No |  |
| Subject | 71.88 | 0.0038 | ** | Yes |  |
|  |  |  |  |  |  |
| ANOVA table | SS | DF | MS | F (DFn, DFd) | P value |
| Group x Time | 216720 | 1 | 216720 | F (1, 24) = 3.427 | P=0.0765 |
| Group | 45786 | 1 | 45786 | F (1, 24) = 0.2340 | P=0.6329 |
| Time | 56958 | 1 | 56958 | F (1, 24) = 0.9008 | P=0.3520 |
| Subject | 4695504 | 24 | 195646 | F (24, 24) = 3.094 | P=0.0038 |
| Residual | 1517551 | 24 | 63231 |  |  |
|  |  |  |  |  |  |
| Difference between row means |  |  |  |  |  |
| Mean of NaCl | 564.0 |  |  |  |  |
| Mean of Cetylpyridinium | 504.7 |  |  |  |  |
| Difference between means | 59.35 |  |  |  |  |
| SE of difference | 122.7 |  |  |  |  |
| 95% CI of difference | -193.8 to 312.5 |  |  |  |  |
|  |  |  |  |  |  |
| Difference between column means |  |  |  |  |  |
| Mean of Pre | 501.2 |  |  |  |  |
| Mean of Post | 567.4 |  |  |  |  |
| Difference between means | -66.19 |  |  |  |  |
| SE of difference | 69.74 |  |  |  |  |
| 95% CI of difference | -210.1 to 77.75 |  |  |  |  |
|  |  |  |  |  |  |
| Interaction CI |  |  |  |  |  |
| Mean diff, A1 - B1 | 62.92 |  |  |  |  |
| Mean diff, A2 - B2 | -195.3 |  |  |  |  |
| (A1 -B1) - (A2 - B2) | 258.2 |  |  |  |  |
| 95% CI of difference | -29.65 to 546.1 |  |  |  |  |
| (B1 - A1) - (B2 - A2) | -258.2 |  |  |  |  |
| 95% CI of difference | -546.1 to 29.65 |  |  |  |  |
|  |  |  |  |  |  |
| Data summary |  |  |  |  |  |
| Number of columns (Time) | 2 |  |  |  |  |
| Number of rows (Group) | 2 |  |  |  |  |
| Number of subjects (Subject) | 26 |  |  |  |  |
| Number of missing values | 0 |  |  |  |  |

| Compare each cell mean with the other cell mean in that row |  |  |  |  |  |  |  |  |
| --- | --- | --- | --- | --- | --- | --- | --- | --- |
|  |  |  |  |  |  |  |  |  |
| Number of families | 1 |  |  |  |  |  |  |  |
| Number of comparisons per family | 2 |  |  |  |  |  |  |  |
| Alpha | 0.05 |  |  |  |  |  |  |  |
|  |  |  |  |  |  |  |  |  |
| Holm-Šídák's multiple comparisons test | Mean Diff. | Below threshold? | Summary | Adjusted P Value |  |  |  |  |
|  |  |  |  |  |  |  |  |  |
| Pre - Post |  |  |  |  |  |  |  |  |
| NaCl | 62.92 | No | ns | 0.5295 |  |  |  |  |
| Cetylpyridinium | -195.3 | No | ns | 0.1150 |  |  |  |  |
|  |  |  |  |  |  |  |  |  |
|  |  |  |  |  |  |  |  |  |
| Test details | Mean 1 | Mean 2 | Mean Diff. | SE of diff. | N1 | N2 | t | DF |
|  |  |  |  |  |  |  |  |  |
| Pre - Post |  |  |  |  |  |  |  |  |
| NaCl | 595.5 | 532.5 | 62.92 | 98.63 | 13 | 13 | 0.6380 | 24.00 |
| Cetylpyridinium | 407.0 | 602.3 | -195.3 | 98.63 | 13 | 13 | 1.980 | 24.00 |

| Table Analyzed | nitrite |  |  |  |  |
| --- | --- | --- | --- | --- | --- |
|  |  |  |  |  |  |
| Two-way RM ANOVA | Matching: Across row |  |  |  |  |
| Assume sphericity? | Yes |  |  |  |  |
| Alpha | 0.05 |  |  |  |  |
|  |  |  |  |  |  |
| Source of Variation | % of total variation | P value | P value summary | Significant? |  |
| Group x Time | 0.2012 | 0.4839 | ns | No |  |
| Group | 0.07368 | 0.8896 | ns | No |  |
| Time | 0.2642 | 0.4232 | ns | No |  |
| Subject | 89.91 | <0.0001 | **** | Yes |  |
|  |  |  |  |  |  |
| ANOVA table | SS | DF | MS | F (DFn, DFd) | P value |
| Group x Time | 1044 | 1 | 1044 | F (1, 24) = 0.5056 | P=0.4839 |
| Group | 382.3 | 1 | 382.3 | F (1, 24) = 0.01967 | P=0.8896 |
| Time | 1371 | 1 | 1371 | F (1, 24) = 0.6640 | P=0.4232 |
| Subject | 466539 | 24 | 19439 | F (24, 24) = 9.415 | P<0.0001 |
| Residual | 49555 | 24 | 2065 |  |  |
|  |  |  |  |  |  |
| Difference between row means |  |  |  |  |  |
| Mean of NaCl | 135.2 |  |  |  |  |
| Mean of Cetylpyridinium | 129.7 |  |  |  |  |
| Difference between means | 5.423 |  |  |  |  |
| SE of difference | 38.67 |  |  |  |  |
| 95% CI of difference | -74.39 to 85.23 |  |  |  |  |
|  |  |  |  |  |  |
| Difference between column means |  |  |  |  |  |
| Mean of Pre | 127.3 |  |  |  |  |
| Mean of Post | 137.6 |  |  |  |  |
| Difference between means | -10.27 |  |  |  |  |
| SE of difference | 12.60 |  |  |  |  |
| 95% CI of difference | -36.28 to 15.74 |  |  |  |  |
|  |  |  |  |  |  |
| Interaction CI |  |  |  |  |  |
| Mean diff, A1 - B1 | -19.23 |  |  |  |  |
| Mean diff, A2 - B2 | -1.308 |  |  |  |  |
| (A1 -B1) - (A2 - B2) | -17.92 |  |  |  |  |
| 95% CI of difference | -69.94 to 34.10 |  |  |  |  |
| (B1 - A1) - (B2 - A2) | 17.92 |  |  |  |  |
| 95% CI of difference | -34.10 to 69.94 |  |  |  |  |
|  |  |  |  |  |  |
| Data summary |  |  |  |  |  |
| Number of columns (Time) | 2 |  |  |  |  |
| Number of rows (Group) | 2 |  |  |  |  |
| Number of subjects (Subject) | 26 |  |  |  |  |
| Number of missing values | 0 |  |  |  |  |

| Compare each cell mean with the other cell mean in that row |  |  |  |  |  |  |  |  |
| --- | --- | --- | --- | --- | --- | --- | --- | --- |
|  |  |  |  |  |  |  |  |  |
| Number of families | 1 |  |  |  |  |  |  |  |
| Number of comparisons per family | 2 |  |  |  |  |  |  |  |
| Alpha | 0.05 |  |  |  |  |  |  |  |
|  |  |  |  |  |  |  |  |  |
| Holm-Šídák's multiple comparisons test | Mean Diff. | Below threshold? | Summary | Adjusted P Value |  |  |  |  |
|  |  |  |  |  |  |  |  |  |
| Pre - Post |  |  |  |  |  |  |  |  |
| NaCl | -19.23 | No | ns | 0.4978 |  |  |  |  |
| Cetylpyridinium | -1.308 | No | ns | 0.9421 |  |  |  |  |
|  |  |  |  |  |  |  |  |  |
|  |  |  |  |  |  |  |  |  |
| Test details | Mean 1 | Mean 2 | Mean Diff. | SE of diff. | N1 | N2 | t | DF |
|  |  |  |  |  |  |  |  |  |
| Pre - Post |  |  |  |  |  |  |  |  |
| NaCl | 125.5 | 144.8 | -19.23 | 17.82 | 13 | 13 | 1.079 | 24.00 |
| Cetylpyridinium | 129.1 | 130.4 | -1.308 | 17.82 | 13 | 13 | 0.07337 | 24.00 |

| Table Analyzed | % nitrite |  |  |  |  |
| --- | --- | --- | --- | --- | --- |
|  |  |  |  |  |  |
| Two-way RM ANOVA | Matching: Across row |  |  |  |  |
| Assume sphericity? | Yes |  |  |  |  |
| Alpha | 0.05 |  |  |  |  |
|  |  |  |  |  |  |
| Source of Variation | % of total variation | P value | P value summary | Significant? |  |
| Group x Time | 5.988 | 0.0038 | ** | Yes |  |
| Group | 0.4777 | 0.7044 | ns | No |  |
| Time | 1.807 | 0.0910 | ns | No |  |
| Subject | 77.74 | <0.0001 | **** | Yes |  |
|  |  |  |  |  |  |
| ANOVA table | SS | DF | MS | F (DFn, DFd) | P value |
| Group x Time | 350.0 | 1 | 350.0 | F (1, 24) = 10.28 | P=0.0038 |
| Group | 27.92 | 1 | 27.92 | F (1, 24) = 0.1475 | P=0.7044 |
| Time | 105.6 | 1 | 105.6 | F (1, 24) = 3.100 | P=0.0910 |
| Subject | 4543 | 24 | 189.3 | F (24, 24) = 5.559 | P<0.0001 |
| Residual | 817.4 | 24 | 34.06 |  |  |
|  |  |  |  |  |  |
| Difference between row means |  |  |  |  |  |
| Mean of NaCl | 19.45 |  |  |  |  |
| Mean of Cetylpyridinium | 20.92 |  |  |  |  |
| Difference between means | -1.465 |  |  |  |  |
| SE of difference | 3.816 |  |  |  |  |
| 95% CI of difference | -9.341 to 6.411 |  |  |  |  |
|  |  |  |  |  |  |
| Difference between column means |  |  |  |  |  |
| Mean of Pre | 21.61 |  |  |  |  |
| Mean of Post | 18.76 |  |  |  |  |
| Difference between means | 2.850 |  |  |  |  |
| SE of difference | 1.619 |  |  |  |  |
| 95% CI of difference | -0.4906 to 6.191 |  |  |  |  |
|  |  |  |  |  |  |
| Interaction CI |  |  |  |  |  |
| Mean diff, A1 - B1 | -2.338 |  |  |  |  |
| Mean diff, A2 - B2 | 8.038 |  |  |  |  |
| (A1 -B1) - (A2 - B2) | -10.38 |  |  |  |  |
| 95% CI of difference | -17.06 to -3.696 |  |  |  |  |
| (B1 - A1) - (B2 - A2) | 10.38 |  |  |  |  |
| 95% CI of difference | 3.696 to 17.06 |  |  |  |  |
|  |  |  |  |  |  |
| Data summary |  |  |  |  |  |
| Number of columns (Time) | 2 |  |  |  |  |
| Number of rows (Group) | 2 |  |  |  |  |
| Number of subjects (Subject) | 26 |  |  |  |  |
| Number of missing values | 0 |  |  |  |  |

| Compare each cell mean with the other cell mean in that row |  |  |  |  |  |  |  |  |
| --- | --- | --- | --- | --- | --- | --- | --- | --- |
|  |  |  |  |  |  |  |  |  |
| Number of families | 1 |  |  |  |  |  |  |  |
| Number of comparisons per family | 2 |  |  |  |  |  |  |  |
| Alpha | 0.05 |  |  |  |  |  |  |  |
|  |  |  |  |  |  |  |  |  |
| Holm-Šídák's multiple comparisons test | Mean Diff. | Below threshold? | Summary | Adjusted P Value |  |  |  |  |
|  |  |  |  |  |  |  |  |  |
| Pre - Post |  |  |  |  |  |  |  |  |
| NaCl | -2.338 | No | ns | 0.3172 |  |  |  |  |
| Cetylpyridinium | 8.038 | Yes | ** | 0.0036 |  |  |  |  |
|  |  |  |  |  |  |  |  |  |
|  |  |  |  |  |  |  |  |  |
| Test details | Mean 1 | Mean 2 | Mean Diff. | SE of diff. | N1 | N2 | t | DF |
|  |  |  |  |  |  |  |  |  |
| Pre - Post |  |  |  |  |  |  |  |  |
| NaCl | 18.28 | 20.62 | -2.338 | 2.289 | 13 | 13 | 1.022 | 24.00 |
| Cetylpyridinium | 24.94 | 16.90 | 8.038 | 2.289 | 13 | 13 | 3.512 | 24.00 |

| Table Analyzed | cGMP |  |  |  |  |
| --- | --- | --- | --- | --- | --- |
|  |  |  |  |  |  |
| Two-way RM ANOVA | Matching: Across row |  |  |  |  |
| Assume sphericity? | Yes |  |  |  |  |
| Alpha | 0.05 |  |  |  |  |
|  |  |  |  |  |  |
| Source of Variation | % of total variation | P value | P value summary | Significant? |  |
| Group x Time | 0.005518 | 0.9317 | ns | No |  |
| Group | 12.20 | 0.0641 | ns | No |  |
| Time | 1.024 | 0.2501 | ns | No |  |
| Subject | 70.63 | 0.0005 | *** | Yes |  |
|  |  |  |  |  |  |
| ANOVA table | SS | DF | MS | F (DFn, DFd) | P value |
| Group x Time | 0.0001910 | 1 | 0.0001910 | F (1, 22) = 0.007517 | P=0.9317 |
| Group | 0.4223 | 1 | 0.4223 | F (1, 22) = 3.800 | P=0.0641 |
| Time | 0.03545 | 1 | 0.03545 | F (1, 22) = 1.395 | P=0.2501 |
| Subject | 2.445 | 22 | 0.1111 | F (22, 22) = 4.374 | P=0.0005 |
| Residual | 0.5590 | 22 | 0.02541 |  |  |
|  |  |  |  |  |  |
| Difference between row means |  |  |  |  |  |
| Mean of NaCl | 0.2011 |  |  |  |  |
| Mean of Cetylpyridinium | 0.3894 |  |  |  |  |
| Difference between means | -0.1882 |  |  |  |  |
| SE of difference | 0.09657 |  |  |  |  |
| 95% CI of difference | -0.3885 to 0.01203 |  |  |  |  |
|  |  |  |  |  |  |
| Difference between column means |  |  |  |  |  |
| Mean of Pre | 0.3225 |  |  |  |  |
| Mean of Post | 0.2680 |  |  |  |  |
| Difference between means | 0.05454 |  |  |  |  |
| SE of difference | 0.04618 |  |  |  |  |
| 95% CI of difference | -0.04122 to 0.1503 |  |  |  |  |
|  |  |  |  |  |  |
| Interaction CI |  |  |  |  |  |
| Mean diff, A1 - B1 | 0.05054 |  |  |  |  |
| Mean diff, A2 - B2 | 0.05855 |  |  |  |  |
| (A1 -B1) - (A2 - B2) | -0.008007 |  |  |  |  |
| 95% CI of difference | -0.1995 to 0.1835 |  |  |  |  |
| (B1 - A1) - (B2 - A2) | 0.008007 |  |  |  |  |
| 95% CI of difference | -0.1835 to 0.1995 |  |  |  |  |
|  |  |  |  |  |  |
| Data summary |  |  |  |  |  |
| Number of columns (Time) | 2 |  |  |  |  |
| Number of rows (Group) | 2 |  |  |  |  |
| Number of subjects (Subject) | 24 |  |  |  |  |
| Number of missing values | 0 |  |  |  |  |

| Compare each cell mean with the other cell mean in that row |  |  |  |  |  |  |  |  |
| --- | --- | --- | --- | --- | --- | --- | --- | --- |
|  |  |  |  |  |  |  |  |  |
| Number of families | 1 |  |  |  |  |  |  |  |
| Number of comparisons per family | 2 |  |  |  |  |  |  |  |
| Alpha | 0.05 |  |  |  |  |  |  |  |
|  |  |  |  |  |  |  |  |  |
| Šídák's multiple comparisons test | Predicted (LS) mean diff. | 95.00% CI of diff. | Below threshold? | Summary | Adjusted P Value |  |  |  |
|  |  |  |  |  |  |  |  |  |
| Pre - Post |  |  |  |  |  |  |  |  |
| NaCl | 0.05054 | -0.09948 to 0.2006 | No | ns | 0.6723 |  |  |  |
| Cetylpyridinium | 0.05855 | -0.1045 to 0.2216 | No | ns | 0.6380 |  |  |  |
|  |  |  |  |  |  |  |  |  |
|  |  |  |  |  |  |  |  |  |
| Test details | Predicted (LS) mean 1 | Predicted (LS) mean 2 | Predicted (LS) mean diff. | SE of diff. | N1 | N2 | t | DF |
|  |  |  |  |  |  |  |  |  |
| Pre - Post |  |  |  |  |  |  |  |  |
| NaCl | 0.2264 | 0.1758 | 0.05054 | 0.06252 | 13 | 13 | 0.8083 | 22.00 |
| Cetylpyridinium | 0.4186 | 0.3601 | 0.05855 | 0.06797 | 11 | 11 | 0.8614 | 22.00 |

| Table Analyzed | breath NO |  |  |  |  |
| --- | --- | --- | --- | --- | --- |
|  |  |  |  |  |  |
| Two-way RM ANOVA | Matching: Across row |  |  |  |  |
| Assume sphericity? | Yes |  |  |  |  |
| Alpha | 0.05 |  |  |  |  |
|  |  |  |  |  |  |
| Source of Variation | % of total variation | P value | P value summary | Significant? |  |
| Group x Time | 0.005412 | 0.9011 | ns | No |  |
| Group | 3.658 | 0.4669 | ns | No |  |
| Time | 0.02165 | 0.8040 | ns | No |  |
| Subject | 91.58 | <0.0001 | **** | Yes |  |
|  |  |  |  |  |  |
| ANOVA table | SS | DF | MS | F (DFn, DFd) | P value |
| Group x Time | 0.1250 | 1 | 0.1250 | F (1, 14) = 0.01600 | P=0.9011 |
| Group | 84.50 | 1 | 84.50 | F (1, 14) = 0.5592 | P=0.4669 |
| Time | 0.5000 | 1 | 0.5000 | F (1, 14) = 0.06400 | P=0.8040 |
| Subject | 2115 | 14 | 151.1 | F (14, 14) = 19.34 | P<0.0001 |
| Residual | 109.4 | 14 | 7.813 |  |  |
|  |  |  |  |  |  |
| Difference between row means |  |  |  |  |  |
| Mean of NaCl | 19.56 |  |  |  |  |
| Mean of Cetylpyridinium | 16.31 |  |  |  |  |
| Difference between means | 3.250 |  |  |  |  |
| SE of difference | 4.346 |  |  |  |  |
| 95% CI of difference | -6.071 to 12.57 |  |  |  |  |
|  |  |  |  |  |  |
| Difference between column means |  |  |  |  |  |
| Mean of Pre | 18.06 |  |  |  |  |
| Mean of Post | 17.81 |  |  |  |  |
| Difference between means | 0.2500 |  |  |  |  |
| SE of difference | 0.9882 |  |  |  |  |
| 95% CI of difference | -1.870 to 2.370 |  |  |  |  |
|  |  |  |  |  |  |
| Interaction CI |  |  |  |  |  |
| Mean diff, A1 - B1 | 0.1250 |  |  |  |  |
| Mean diff, A2 - B2 | 0.3750 |  |  |  |  |
| (A1 -B1) - (A2 - B2) | -0.2500 |  |  |  |  |
| 95% CI of difference | -4.489 to 3.989 |  |  |  |  |
| (B1 - A1) - (B2 - A2) | 0.2500 |  |  |  |  |
| 95% CI of difference | -3.989 to 4.489 |  |  |  |  |
|  |  |  |  |  |  |
| Data summary |  |  |  |  |  |
| Number of columns (Time) | 2 |  |  |  |  |
| Number of rows (Group) | 2 |  |  |  |  |
| Number of subjects (Subject) | 16 |  |  |  |  |
| Number of missing values | 0 |  |  |  |  |

| Compare each cell mean with the other cell mean in that row |  |  |  |  |  |  |  |  |
| --- | --- | --- | --- | --- | --- | --- | --- | --- |
|  |  |  |  |  |  |  |  |  |
| Number of families | 1 |  |  |  |  |  |  |  |
| Number of comparisons per family | 2 |  |  |  |  |  |  |  |
| Alpha | 0.05 |  |  |  |  |  |  |  |
|  |  |  |  |  |  |  |  |  |
| Šídák's multiple comparisons test | Mean Diff. | 95.00% CI of diff. | Below threshold? | Summary | Adjusted P Value |  |  |  |
|  |  |  |  |  |  |  |  |  |
| Pre - Post |  |  |  |  |  |  |  |  |
| NaCl | 0.1250 | -3.373 to 3.623 | No | ns | 0.9951 |  |  |  |
| Cetylpyridinium | 0.3750 | -3.123 to 3.873 | No | ns | 0.9569 |  |  |  |
|  |  |  |  |  |  |  |  |  |
|  |  |  |  |  |  |  |  |  |
| Test details | Mean 1 | Mean 2 | Mean Diff. | SE of diff. | N1 | N2 | t | DF |
|  |  |  |  |  |  |  |  |  |
| Pre - Post |  |  |  |  |  |  |  |  |
| NaCl | 19.63 | 19.50 | 0.1250 | 1.398 | 8 | 8 | 0.08944 | 14.00 |
| Cetylpyridinium | 16.50 | 16.13 | 0.3750 | 1.398 | 8 | 8 | 0.2683 | 14.00 |

| Table Analyzed | peak torque |  |  |  |  |
| --- | --- | --- | --- | --- | --- |
|  |  |  |  |  |  |
| Three-way ANOVA | Matching by factors: Velocity & Time |  |  |  |  |
| Assume sphericity? | No |  |  |  |  |
| Alpha | 0.05 |  |  |  |  |
|  |  |  |  |  |  |
| Source of Variation | % of total variation | P value | P value summary | Significant? | Geisser-Greenhouse's epsilon |
| Velocity | 50.66 | <0.0001 | **** | Yes | 0.4018 |
| Group | 0.1781 | 0.7095 | ns | No |  |
| Time | 0.006337 | 0.6427 | ns | No | 1.000 |
| Velocity x Group | 0.2328 | 0.6799 | ns | No |  |
| Velocity x Time | 0.03448 | 0.4975 | ns | No | 0.4327 |
| Group x Time | 0.08025 | 0.1063 | ns | No |  |
| Velocity x Group x Time | 0.02598 | 0.7344 | ns | No |  |
| Subject | 35.22 |  |  |  |  |
| Subject x Velocity | 11.30 |  |  |  |  |
| Subject x Time | 0.8067 |  |  |  |  |
|  |  |  |  |  |  |
| ANOVA table | SS | DF | MS | F (DFn, DFd) | P value |
| Velocity | 85.39 | 4 | 21.35 | F (1.607, 45.00) = 125.5 | P<0.0001 |
| Group | 0.3002 | 1 | 0.3002 | F (1, 28) = 0.1416 | P=0.7095 |
| Time | 0.01068 | 1 | 0.01068 | F (1.000, 28.00) = 0.2199 | P=0.6427 |
| Velocity x Group | 0.3924 | 4 | 0.09811 | F (4, 112) = 0.5770 | P=0.6799 |
| Velocity x Time | 0.05810 | 4 | 0.01453 | F (1.731, 48.46) = 0.6661 | P=0.4975 |
| Group x Time | 0.1353 | 1 | 0.1353 | F (1, 28) = 2.785 | P=0.1063 |
| Velocity x Group x Time | 0.04378 | 4 | 0.01095 | F (4, 112) = 0.5019 | P=0.7344 |
| Subject | 59.36 | 28 | 2.120 |  |  |
| Subject x Velocity | 19.04 | 112 | 0.1700 |  |  |
| Subject x Time | 1.360 | 28 | 0.04856 |  |  |
| Residual | 2.443 | 112 | 0.02181 |  |  |
|  |  |  |  |  |  |
| Data summary |  |  |  |  |  |
| Number of columns | 2 x 2 |  |  |  |  |
| Number of rows (Velocity) | 5 |  |  |  |  |
| Number of subjects (Subject) | 30 |  |  |  |  |
| Number of missing values | 0 |  |  |  |  |

| Compare each cell mean with every other cell mean |  |  |  |  |  |  |  |  |
| --- | --- | --- | --- | --- | --- | --- | --- | --- |
|  |  |  |  |  |  |  |  |  |
| Number of families | 1 |  |  |  |  |  |  |  |
| Number of comparisons per family | 190 |  |  |  |  |  |  |  |
| Alpha | 0.05 |  |  |  |  |  |  |  |
|  |  |  |  |  |  |  |  |  |
| Holm-Šídák's multiple comparisons test | Mean Diff. | Below threshold? | Summary | Adjusted P Value |  |  |  |  |
|  |  |  |  |  |  |  |  |  |
| 0:NaCl - pre vs. 0:NaCl - post | -0.09000 | No | ns | 0.9991 |  |  |  |  |
| 0:NaCl - pre vs. 0:Cetylpyridinium - pre | 0.1400 | No | ns | >0.9999 |  |  |  |  |
| 0:NaCl - pre vs. 0:Cetylpyridinium - post | 0.1853 | No | ns | >0.9999 |  |  |  |  |
| 0:NaCl - pre vs. 1.57:NaCl - pre | 0.5993 | Yes | * | 0.0325 |  |  |  |  |
| 0:NaCl - pre vs. 1.57:NaCl - post | 0.5127 | Yes | * | 0.0284 |  |  |  |  |
| 0:NaCl - pre vs. 1.57:Cetylpyridinium - pre | 0.5640 | No | ns | 0.6276 |  |  |  |  |
| 0:NaCl - pre vs. 1.57:Cetylpyridinium - post | 0.5973 | No | ns | 0.4444 |  |  |  |  |
| 0:NaCl - pre vs. 3.14:NaCl - pre | 0.9720 | Yes | *** | 0.0003 |  |  |  |  |
| 0:NaCl - pre vs. 3.14:NaCl - post | 0.9520 | Yes | *** | 0.0002 |  |  |  |  |
| 0:NaCl - pre vs. 3.14:Cetylpyridinium - pre | 0.9467 | Yes | ** | 0.0082 |  |  |  |  |
| 0:NaCl - pre vs. 3.14:Cetylpyridinium - post | 1.043 | Yes | ** | 0.0021 |  |  |  |  |
| 0:NaCl - pre vs. 4.71:NaCl - pre | 1.300 | Yes | **** | <0.0001 |  |  |  |  |
| 0:NaCl - pre vs. 4.71:NaCl - post | 1.277 | Yes | **** | <0.0001 |  |  |  |  |
| 0:NaCl - pre vs. 4.71:Cetylpyridinium - pre | 1.313 | Yes | **** | <0.0001 |  |  |  |  |
| 0:NaCl - pre vs. 4.71:Cetylpyridinium - post | 1.326 | Yes | **** | <0.0001 |  |  |  |  |
| 0:NaCl - pre vs. 6.28:NaCl - pre | 1.577 | Yes | **** | <0.0001 |  |  |  |  |
| 0:NaCl - pre vs. 6.28:NaCl - post | 1.524 | Yes | **** | <0.0001 |  |  |  |  |
| 0:NaCl - pre vs. 6.28:Cetylpyridinium - pre | 1.588 | Yes | **** | <0.0001 |  |  |  |  |
| 0:NaCl - pre vs. 6.28:Cetylpyridinium - post | 1.553 | Yes | **** | <0.0001 |  |  |  |  |
| 0:NaCl - post vs. 0:Cetylpyridinium - pre | 0.2300 | No | ns | >0.9999 |  |  |  |  |
| 0:NaCl - post vs. 0:Cetylpyridinium - post | 0.2753 | No | ns | 0.9999 |  |  |  |  |
| 0:NaCl - post vs. 1.57:NaCl - pre | 0.6893 | Yes | * | 0.0363 |  |  |  |  |
| 0:NaCl - post vs. 1.57:NaCl - post | 0.6027 | Yes | * | 0.0259 |  |  |  |  |
| 0:NaCl - post vs. 1.57:Cetylpyridinium - pre | 0.6540 | No | ns | 0.3461 |  |  |  |  |
| 0:NaCl - post vs. 1.57:Cetylpyridinium - post | 0.6873 | No | ns | 0.2233 |  |  |  |  |
| 0:NaCl - post vs. 3.14:NaCl - pre | 1.062 | Yes | *** | 0.0002 |  |  |  |  |
| 0:NaCl - post vs. 3.14:NaCl - post | 1.042 | Yes | *** | 0.0002 |  |  |  |  |
| 0:NaCl - post vs. 3.14:Cetylpyridinium - pre | 1.037 | Yes | ** | 0.0033 |  |  |  |  |
| 0:NaCl - post vs. 3.14:Cetylpyridinium - post | 1.133 | Yes | *** | 0.0008 |  |  |  |  |
| 0:NaCl - post vs. 4.71:NaCl - pre | 1.390 | Yes | **** | <0.0001 |  |  |  |  |
| 0:NaCl - post vs. 4.71:NaCl - post | 1.367 | Yes | **** | <0.0001 |  |  |  |  |
| 0:NaCl - post vs. 4.71:Cetylpyridinium - pre | 1.403 | Yes | **** | <0.0001 |  |  |  |  |
| 0:NaCl - post vs. 4.71:Cetylpyridinium - post | 1.416 | Yes | **** | <0.0001 |  |  |  |  |
| 0:NaCl - post vs. 6.28:NaCl - pre | 1.667 | Yes | **** | <0.0001 |  |  |  |  |
| 0:NaCl - post vs. 6.28:NaCl - post | 1.614 | Yes | **** | <0.0001 |  |  |  |  |
| 0:NaCl - post vs. 6.28:Cetylpyridinium - pre | 1.678 | Yes | **** | <0.0001 |  |  |  |  |
| 0:NaCl - post vs. 6.28:Cetylpyridinium - post | 1.643 | Yes | **** | <0.0001 |  |  |  |  |
| 0:Cetylpyridinium - pre vs. 0:Cetylpyridinium - post | 0.04533 | No | ns | >0.9999 |  |  |  |  |
| 0:Cetylpyridinium - pre vs. 1.57:NaCl - pre | 0.4593 | No | ns | 0.9729 |  |  |  |  |
| 0:Cetylpyridinium - pre vs. 1.57:NaCl - post | 0.3727 | No | ns | 0.9960 |  |  |  |  |
| 0:Cetylpyridinium - pre vs. 1.57:Cetylpyridinium - pre | 0.4240 | No | ns | 0.9619 |  |  |  |  |
| 0:Cetylpyridinium - pre vs. 1.57:Cetylpyridinium - post | 0.4573 | No | ns | 0.8942 |  |  |  |  |
| 0:Cetylpyridinium - pre vs. 3.14:NaCl - pre | 0.8320 | No | ns | 0.1549 |  |  |  |  |
| 0:Cetylpyridinium - pre vs. 3.14:NaCl - post | 0.8120 | No | ns | 0.1596 |  |  |  |  |
| 0:Cetylpyridinium - pre vs. 3.14:Cetylpyridinium - pre | 0.8067 | No | ns | 0.0974 |  |  |  |  |
| 0:Cetylpyridinium - pre vs. 3.14:Cetylpyridinium - post | 0.9027 | No | ns | 0.0616 |  |  |  |  |
| 0:Cetylpyridinium - pre vs. 4.71:NaCl - pre | 1.160 | Yes | ** | 0.0075 |  |  |  |  |
| 0:Cetylpyridinium - pre vs. 4.71:NaCl - post | 1.137 | Yes | * | 0.0105 |  |  |  |  |
| 0:Cetylpyridinium - pre vs. 4.71:Cetylpyridinium - pre | 1.173 | Yes | ** | 0.0035 |  |  |  |  |
| 0:Cetylpyridinium - pre vs. 4.71:Cetylpyridinium - post | 1.186 | Yes | ** | 0.0031 |  |  |  |  |
| 0:Cetylpyridinium - pre vs. 6.28:NaCl - pre | 1.437 | Yes | *** | 0.0006 |  |  |  |  |
| 0:Cetylpyridinium - pre vs. 6.28:NaCl - post | 1.384 | Yes | ** | 0.0011 |  |  |  |  |
| 0:Cetylpyridinium - pre vs. 6.28:Cetylpyridinium - pre | 1.448 | Yes | *** | 0.0002 |  |  |  |  |
| 0:Cetylpyridinium - pre vs. 6.28:Cetylpyridinium - post | 1.413 | Yes | *** | 0.0004 |  |  |  |  |
| 0:Cetylpyridinium - post vs. 1.57:NaCl - pre | 0.4140 | No | ns | 0.9875 |  |  |  |  |
| 0:Cetylpyridinium - post vs. 1.57:NaCl - post | 0.3273 | No | ns | 0.9991 |  |  |  |  |
| 0:Cetylpyridinium - post vs. 1.57:Cetylpyridinium - pre | 0.3787 | No | ns | 0.8627 |  |  |  |  |
| 0:Cetylpyridinium - post vs. 1.57:Cetylpyridinium - post | 0.4120 | No | ns | 0.6853 |  |  |  |  |
| 0:Cetylpyridinium - post vs. 3.14:NaCl - pre | 0.7867 | No | ns | 0.2263 |  |  |  |  |
| 0:Cetylpyridinium - post vs. 3.14:NaCl - post | 0.7667 | No | ns | 0.2358 |  |  |  |  |
| 0:Cetylpyridinium - post vs. 3.14:Cetylpyridinium - pre | 0.7613 | Yes | * | 0.0319 |  |  |  |  |
| 0:Cetylpyridinium - post vs. 3.14:Cetylpyridinium - post | 0.8573 | Yes | * | 0.0159 |  |  |  |  |
| 0:Cetylpyridinium - post vs. 4.71:NaCl - pre | 1.115 | Yes | * | 0.0115 |  |  |  |  |
| 0:Cetylpyridinium - post vs. 4.71:NaCl - post | 1.092 | Yes | * | 0.0163 |  |  |  |  |
| 0:Cetylpyridinium - post vs. 4.71:Cetylpyridinium - pre | 1.128 | Yes | *** | 0.0008 |  |  |  |  |
| 0:Cetylpyridinium - post vs. 4.71:Cetylpyridinium - post | 1.141 | Yes | *** | 0.0007 |  |  |  |  |
| 0:Cetylpyridinium - post vs. 6.28:NaCl - pre | 1.391 | Yes | *** | 0.0010 |  |  |  |  |
| 0:Cetylpyridinium - post vs. 6.28:NaCl - post | 1.339 | Yes | ** | 0.0018 |  |  |  |  |
| 0:Cetylpyridinium - post vs. 6.28:Cetylpyridinium - pre | 1.403 | Yes | **** | <0.0001 |  |  |  |  |
| 0:Cetylpyridinium - post vs. 6.28:Cetylpyridinium - post | 1.368 | Yes | **** | <0.0001 |  |  |  |  |
| 1.57:NaCl - pre vs. 1.57:NaCl - post | -0.08667 | No | ns | 0.9990 |  |  |  |  |
| 1.57:NaCl - pre vs. 1.57:Cetylpyridinium - pre | -0.03533 | No | ns | >0.9999 |  |  |  |  |
| 1.57:NaCl - pre vs. 1.57:Cetylpyridinium - post | -0.002000 | No | ns | >0.9999 |  |  |  |  |
| 1.57:NaCl - pre vs. 3.14:NaCl - pre | 0.3727 | Yes | ** | 0.0011 |  |  |  |  |
| 1.57:NaCl - pre vs. 3.14:NaCl - post | 0.3527 | Yes | ** | 0.0038 |  |  |  |  |
| 1.57:NaCl - pre vs. 3.14:Cetylpyridinium - pre | 0.3473 | No | ns | 0.9828 |  |  |  |  |
| 1.57:NaCl - pre vs. 3.14:Cetylpyridinium - post | 0.4433 | No | ns | 0.8627 |  |  |  |  |
| 1.57:NaCl - pre vs. 4.71:NaCl - pre | 0.7007 | Yes | **** | <0.0001 |  |  |  |  |
| 1.57:NaCl - pre vs. 4.71:NaCl - post | 0.6780 | Yes | **** | <0.0001 |  |  |  |  |
| 1.57:NaCl - pre vs. 4.71:Cetylpyridinium - pre | 0.7140 | Yes | * | 0.0421 |  |  |  |  |
| 1.57:NaCl - pre vs. 4.71:Cetylpyridinium - post | 0.7267 | Yes | * | 0.0319 |  |  |  |  |
| 1.57:NaCl - pre vs. 6.28:NaCl - pre | 0.9773 | Yes | **** | <0.0001 |  |  |  |  |
| 1.57:NaCl - pre vs. 6.28:NaCl - post | 0.9247 | Yes | **** | <0.0001 |  |  |  |  |
| 1.57:NaCl - pre vs. 6.28:Cetylpyridinium - pre | 0.9887 | Yes | *** | 0.0007 |  |  |  |  |
| 1.57:NaCl - pre vs. 6.28:Cetylpyridinium - post | 0.9540 | Yes | *** | 0.0007 |  |  |  |  |
| 1.57:NaCl - post vs. 1.57:Cetylpyridinium - pre | 0.05133 | No | ns | >0.9999 |  |  |  |  |
| 1.57:NaCl - post vs. 1.57:Cetylpyridinium - post | 0.08467 | No | ns | >0.9999 |  |  |  |  |
| 1.57:NaCl - post vs. 3.14:NaCl - pre | 0.4593 | Yes | *** | 0.0005 |  |  |  |  |
| 1.57:NaCl - post vs. 3.14:NaCl - post | 0.4393 | Yes | **** | <0.0001 |  |  |  |  |
| 1.57:NaCl - post vs. 3.14:Cetylpyridinium - pre | 0.4340 | No | ns | 0.8942 |  |  |  |  |
| 1.57:NaCl - post vs. 3.14:Cetylpyridinium - post | 0.5300 | No | ns | 0.5459 |  |  |  |  |
| 1.57:NaCl - post vs. 4.71:NaCl - pre | 0.7873 | Yes | **** | <0.0001 |  |  |  |  |
| 1.57:NaCl - post vs. 4.71:NaCl - post | 0.7647 | Yes | **** | <0.0001 |  |  |  |  |
| 1.57:NaCl - post vs. 4.71:Cetylpyridinium - pre | 0.8007 | Yes | * | 0.0135 |  |  |  |  |
| 1.57:NaCl - post vs. 4.71:Cetylpyridinium - post | 0.8133 | Yes | ** | 0.0099 |  |  |  |  |
| 1.57:NaCl - post vs. 6.28:NaCl - pre | 1.064 | Yes | **** | <0.0001 |  |  |  |  |
| 1.57:NaCl - post vs. 6.28:NaCl - post | 1.011 | Yes | **** | <0.0001 |  |  |  |  |
| 1.57:NaCl - post vs. 6.28:Cetylpyridinium - pre | 1.075 | Yes | *** | 0.0002 |  |  |  |  |
| 1.57:NaCl - post vs. 6.28:Cetylpyridinium - post | 1.041 | Yes | *** | 0.0002 |  |  |  |  |
| 1.57:Cetylpyridinium - pre vs. 1.57:Cetylpyridinium - post | 0.03333 | No | ns | >0.9999 |  |  |  |  |
| 1.57:Cetylpyridinium - pre vs. 3.14:NaCl - pre | 0.4080 | No | ns | 0.9611 |  |  |  |  |
| 1.57:Cetylpyridinium - pre vs. 3.14:NaCl - post | 0.3880 | No | ns | 0.9622 |  |  |  |  |
| 1.57:Cetylpyridinium - pre vs. 3.14:Cetylpyridinium - pre | 0.3827 | Yes | **** | <0.0001 |  |  |  |  |
| 1.57:Cetylpyridinium - pre vs. 3.14:Cetylpyridinium - post | 0.4787 | Yes | *** | 0.0002 |  |  |  |  |
| 1.57:Cetylpyridinium - pre vs. 4.71:NaCl - pre | 0.7360 | No | ns | 0.0879 |  |  |  |  |
| 1.57:Cetylpyridinium - pre vs. 4.71:NaCl - post | 0.7133 | No | ns | 0.1438 |  |  |  |  |
| 1.57:Cetylpyridinium - pre vs. 4.71:Cetylpyridinium - pre | 0.7493 | Yes | **** | <0.0001 |  |  |  |  |
| 1.57:Cetylpyridinium - pre vs. 4.71:Cetylpyridinium - post | 0.7620 | Yes | **** | <0.0001 |  |  |  |  |
| 1.57:Cetylpyridinium - pre vs. 6.28:NaCl - pre | 1.013 | Yes | ** | 0.0038 |  |  |  |  |
| 1.57:Cetylpyridinium - pre vs. 6.28:NaCl - post | 0.9600 | Yes | * | 0.0105 |  |  |  |  |
| 1.57:Cetylpyridinium - pre vs. 6.28:Cetylpyridinium - pre | 1.024 | Yes | **** | <0.0001 |  |  |  |  |
| 1.57:Cetylpyridinium - pre vs. 6.28:Cetylpyridinium - post | 0.9893 | Yes | **** | <0.0001 |  |  |  |  |
| 1.57:Cetylpyridinium - post vs. 3.14:NaCl - pre | 0.3747 | No | ns | 0.9729 |  |  |  |  |
| 1.57:Cetylpyridinium - post vs. 3.14:NaCl - post | 0.3547 | No | ns | 0.9729 |  |  |  |  |
| 1.57:Cetylpyridinium - post vs. 3.14:Cetylpyridinium - pre | 0.3493 | Yes | ** | 0.0056 |  |  |  |  |
| 1.57:Cetylpyridinium - post vs. 3.14:Cetylpyridinium - post | 0.4453 | Yes | **** | <0.0001 |  |  |  |  |
| 1.57:Cetylpyridinium - post vs. 4.71:NaCl - pre | 0.7027 | No | ns | 0.0938 |  |  |  |  |
| 1.57:Cetylpyridinium - post vs. 4.71:NaCl - post | 0.6800 | No | ns | 0.1554 |  |  |  |  |
| 1.57:Cetylpyridinium - post vs. 4.71:Cetylpyridinium - pre | 0.7160 | Yes | **** | <0.0001 |  |  |  |  |
| 1.57:Cetylpyridinium - post vs. 4.71:Cetylpyridinium - post | 0.7287 | Yes | **** | <0.0001 |  |  |  |  |
| 1.57:Cetylpyridinium - post vs. 6.28:NaCl - pre | 0.9793 | Yes | ** | 0.0036 |  |  |  |  |
| 1.57:Cetylpyridinium - post vs. 6.28:NaCl - post | 0.9267 | Yes | * | 0.0105 |  |  |  |  |
| 1.57:Cetylpyridinium - post vs. 6.28:Cetylpyridinium - pre | 0.9907 | Yes | **** | <0.0001 |  |  |  |  |
| 1.57:Cetylpyridinium - post vs. 6.28:Cetylpyridinium - post | 0.9560 | Yes | **** | <0.0001 |  |  |  |  |
| 3.14:NaCl - pre vs. 3.14:NaCl - post | -0.02000 | No | ns | >0.9999 |  |  |  |  |
| 3.14:NaCl - pre vs. 3.14:Cetylpyridinium - pre | -0.02533 | No | ns | >0.9999 |  |  |  |  |
| 3.14:NaCl - pre vs. 3.14:Cetylpyridinium - post | 0.07067 | No | ns | >0.9999 |  |  |  |  |
| 3.14:NaCl - pre vs. 4.71:NaCl - pre | 0.3280 | Yes | *** | 0.0003 |  |  |  |  |
| 3.14:NaCl - pre vs. 4.71:NaCl - post | 0.3053 | No | ns | 0.0974 |  |  |  |  |
| 3.14:NaCl - pre vs. 4.71:Cetylpyridinium - pre | 0.3413 | No | ns | 0.9568 |  |  |  |  |
| 3.14:NaCl - pre vs. 4.71:Cetylpyridinium - post | 0.3540 | No | ns | 0.9214 |  |  |  |  |
| 3.14:NaCl - pre vs. 6.28:NaCl - pre | 0.6047 | Yes | *** | 0.0003 |  |  |  |  |
| 3.14:NaCl - pre vs. 6.28:NaCl - post | 0.5520 | Yes | *** | 0.0010 |  |  |  |  |
| 3.14:NaCl - pre vs. 6.28:Cetylpyridinium - pre | 0.6160 | No | ns | 0.0797 |  |  |  |  |
| 3.14:NaCl - pre vs. 6.28:Cetylpyridinium - post | 0.5813 | No | ns | 0.0819 |  |  |  |  |
| 3.14:NaCl - post vs. 3.14:Cetylpyridinium - pre | -0.005333 | No | ns | >0.9999 |  |  |  |  |
| 3.14:NaCl - post vs. 3.14:Cetylpyridinium - post | 0.09067 | No | ns | >0.9999 |  |  |  |  |
| 3.14:NaCl - post vs. 4.71:NaCl - pre | 0.3480 | Yes | *** | 0.0004 |  |  |  |  |
| 3.14:NaCl - post vs. 4.71:NaCl - post | 0.3253 | Yes | *** | 0.0008 |  |  |  |  |
| 3.14:NaCl - post vs. 4.71:Cetylpyridinium - pre | 0.3613 | No | ns | 0.8743 |  |  |  |  |
| 3.14:NaCl - post vs. 4.71:Cetylpyridinium - post | 0.3740 | No | ns | 0.8117 |  |  |  |  |
| 3.14:NaCl - post vs. 6.28:NaCl - pre | 0.6247 | Yes | **** | <0.0001 |  |  |  |  |
| 3.14:NaCl - post vs. 6.28:NaCl - post | 0.5720 | Yes | **** | <0.0001 |  |  |  |  |
| 3.14:NaCl - post vs. 6.28:Cetylpyridinium - pre | 0.6360 | Yes | * | 0.0363 |  |  |  |  |
| 3.14:NaCl - post vs. 6.28:Cetylpyridinium - post | 0.6013 | Yes | * | 0.0341 |  |  |  |  |
| 3.14:Cetylpyridinium - pre vs. 3.14:Cetylpyridinium - post | 0.09600 | No | ns | 0.9619 |  |  |  |  |
| 3.14:Cetylpyridinium - pre vs. 4.71:NaCl - pre | 0.3533 | No | ns | 0.9622 |  |  |  |  |
| 3.14:Cetylpyridinium - pre vs. 4.71:NaCl - post | 0.3307 | No | ns | 0.9828 |  |  |  |  |
| 3.14:Cetylpyridinium - pre vs. 4.71:Cetylpyridinium - pre | 0.3667 | Yes | **** | <0.0001 |  |  |  |  |
| 3.14:Cetylpyridinium - pre vs. 4.71:Cetylpyridinium - post | 0.3793 | Yes | *** | 0.0006 |  |  |  |  |
| 3.14:Cetylpyridinium - pre vs. 6.28:NaCl - pre | 0.6300 | No | ns | 0.1424 |  |  |  |  |
| 3.14:Cetylpyridinium - pre vs. 6.28:NaCl - post | 0.5773 | No | ns | 0.3419 |  |  |  |  |
| 3.14:Cetylpyridinium - pre vs. 6.28:Cetylpyridinium - pre | 0.6413 | Yes | *** | 0.0003 |  |  |  |  |
| 3.14:Cetylpyridinium - pre vs. 6.28:Cetylpyridinium - post | 0.6067 | Yes | *** | 0.0002 |  |  |  |  |
| 3.14:Cetylpyridinium - post vs. 4.71:NaCl - pre | 0.2573 | No | ns | 0.9977 |  |  |  |  |
| 3.14:Cetylpyridinium - post vs. 4.71:NaCl - post | 0.2347 | No | ns | 0.9995 |  |  |  |  |
| 3.14:Cetylpyridinium - post vs. 4.71:Cetylpyridinium - pre | 0.2707 | Yes | * | 0.0136 |  |  |  |  |
| 3.14:Cetylpyridinium - post vs. 4.71:Cetylpyridinium - post | 0.2833 | Yes | *** | 0.0003 |  |  |  |  |
| 3.14:Cetylpyridinium - post vs. 6.28:NaCl - pre | 0.5340 | No | ns | 0.3419 |  |  |  |  |
| 3.14:Cetylpyridinium - post vs. 6.28:NaCl - post | 0.4813 | No | ns | 0.6724 |  |  |  |  |
| 3.14:Cetylpyridinium - post vs. 6.28:Cetylpyridinium - pre | 0.5453 | Yes | ** | 0.0079 |  |  |  |  |
| 3.14:Cetylpyridinium - post vs. 6.28:Cetylpyridinium - post | 0.5107 | Yes | *** | 0.0004 |  |  |  |  |
| 4.71:NaCl - pre vs. 4.71:NaCl - post | -0.02267 | No | ns | >0.9999 |  |  |  |  |
| 4.71:NaCl - pre vs. 4.71:Cetylpyridinium - pre | 0.01333 | No | ns | >0.9999 |  |  |  |  |
| 4.71:NaCl - pre vs. 4.71:Cetylpyridinium - post | 0.02600 | No | ns | >0.9999 |  |  |  |  |
| 4.71:NaCl - pre vs. 6.28:NaCl - pre | 0.2767 | Yes | ** | 0.0027 |  |  |  |  |
| 4.71:NaCl - pre vs. 6.28:NaCl - post | 0.2240 | No | ns | 0.0704 |  |  |  |  |
| 4.71:NaCl - pre vs. 6.28:Cetylpyridinium - pre | 0.2880 | No | ns | 0.9729 |  |  |  |  |
| 4.71:NaCl - pre vs. 6.28:Cetylpyridinium - post | 0.2533 | No | ns | 0.9828 |  |  |  |  |
| 4.71:NaCl - post vs. 4.71:Cetylpyridinium - pre | 0.03600 | No | ns | >0.9999 |  |  |  |  |
| 4.71:NaCl - post vs. 4.71:Cetylpyridinium - post | 0.04867 | No | ns | >0.9999 |  |  |  |  |
| 4.71:NaCl - post vs. 6.28:NaCl - pre | 0.2993 | Yes | ** | 0.0031 |  |  |  |  |
| 4.71:NaCl - post vs. 6.28:NaCl - post | 0.2467 | Yes | **** | <0.0001 |  |  |  |  |
| 4.71:NaCl - post vs. 6.28:Cetylpyridinium - pre | 0.3107 | No | ns | 0.9729 |  |  |  |  |
| 4.71:NaCl - post vs. 6.28:Cetylpyridinium - post | 0.2760 | No | ns | 0.9828 |  |  |  |  |
| 4.71:Cetylpyridinium - pre vs. 4.71:Cetylpyridinium - post | 0.01267 | No | ns | >0.9999 |  |  |  |  |
| 4.71:Cetylpyridinium - pre vs. 6.28:NaCl - pre | 0.2633 | No | ns | 0.9875 |  |  |  |  |
| 4.71:Cetylpyridinium - pre vs. 6.28:NaCl - post | 0.2107 | No | ns | 0.9995 |  |  |  |  |
| 4.71:Cetylpyridinium - pre vs. 6.28:Cetylpyridinium - pre | 0.2747 | Yes | * | 0.0456 |  |  |  |  |
| 4.71:Cetylpyridinium - pre vs. 6.28:Cetylpyridinium - post | 0.2400 | Yes | * | 0.0456 |  |  |  |  |
| 4.71:Cetylpyridinium - post vs. 6.28:NaCl - pre | 0.2507 | No | ns | 0.9906 |  |  |  |  |
| 4.71:Cetylpyridinium - post vs. 6.28:NaCl - post | 0.1980 | No | ns | 0.9997 |  |  |  |  |
| 4.71:Cetylpyridinium - post vs. 6.28:Cetylpyridinium - pre | 0.2620 | No | ns | 0.1451 |  |  |  |  |
| 4.71:Cetylpyridinium - post vs. 6.28:Cetylpyridinium - post | 0.2273 | Yes | ** | 0.0029 |  |  |  |  |
| 6.28:NaCl - pre vs. 6.28:NaCl - post | -0.05267 | No | ns | 0.9995 |  |  |  |  |
| 6.28:NaCl - pre vs. 6.28:Cetylpyridinium - pre | 0.01133 | No | ns | >0.9999 |  |  |  |  |
| 6.28:NaCl - pre vs. 6.28:Cetylpyridinium - post | -0.02333 | No | ns | >0.9999 |  |  |  |  |
| 6.28:NaCl - post vs. 6.28:Cetylpyridinium - pre | 0.06400 | No | ns | >0.9999 |  |  |  |  |
| 6.28:NaCl - post vs. 6.28:Cetylpyridinium - post | 0.02933 | No | ns | >0.9999 |  |  |  |  |
| 6.28:Cetylpyridinium - pre vs. 6.28:Cetylpyridinium - post | -0.03467 | No | ns | >0.9999 |  |  |  |  |
|  |  |  |  |  |  |  |  |  |
|  |  |  |  |  |  |  |  |  |

| Test details | Mean 1 | Mean 2 | Mean Diff. | SE of diff. | N1 | N2 | t | DF |
| --- | --- | --- | --- | --- | --- | --- | --- | --- |
|  |  |  |  |  |  |  |  |  |
| 0:NaCl - pre vs. 0:NaCl - post | 2.651 | 2.741 | -0.09000 | 0.06644 | 15 | 15 | 1.355 | 14.00 |
| 0:NaCl - pre vs. 0:Cetylpyridinium - pre | 2.651 | 2.511 | 0.1400 | 0.2506 | 15 | 15 | 0.5586 | 24.16 |
| 0:NaCl - pre vs. 0:Cetylpyridinium - post | 2.651 | 2.465 | 0.1853 | 0.2514 | 15 | 15 | 0.7372 | 24.10 |
| 0:NaCl - pre vs. 1.57:NaCl - pre | 2.651 | 2.051 | 0.5993 | 0.1282 | 15 | 15 | 4.674 | 14.00 |
| 0:NaCl - pre vs. 1.57:NaCl - post | 2.651 | 2.138 | 0.5127 | 0.1077 | 15 | 15 | 4.762 | 14.00 |
| 0:NaCl - pre vs. 1.57:Cetylpyridinium - pre | 2.651 | 2.087 | 0.5640 | 0.2183 | 15 | 15 | 2.583 | 26.84 |
| 0:NaCl - pre vs. 1.57:Cetylpyridinium - post | 2.651 | 2.053 | 0.5973 | 0.2124 | 15 | 15 | 2.813 | 27.28 |
| 0:NaCl - pre vs. 3.14:NaCl - pre | 2.651 | 1.679 | 0.9720 | 0.1280 | 15 | 15 | 7.596 | 14.00 |
| 0:NaCl - pre vs. 3.14:NaCl - post | 2.651 | 1.699 | 0.9520 | 0.1195 | 15 | 15 | 7.968 | 14.00 |
| 0:NaCl - pre vs. 3.14:Cetylpyridinium - pre | 2.651 | 1.704 | 0.9467 | 0.2045 | 15 | 15 | 4.630 | 27.74 |
| 0:NaCl - pre vs. 3.14:Cetylpyridinium - post | 2.651 | 1.608 | 1.043 | 0.2014 | 15 | 15 | 5.177 | 27.87 |
| 0:NaCl - pre vs. 4.71:NaCl - pre | 2.651 | 1.351 | 1.300 | 0.1367 | 15 | 15 | 9.512 | 14.00 |
| 0:NaCl - pre vs. 4.71:NaCl - post | 2.651 | 1.373 | 1.277 | 0.1456 | 15 | 15 | 8.775 | 14.00 |
| 0:NaCl - pre vs. 4.71:Cetylpyridinium - pre | 2.651 | 1.337 | 1.313 | 0.1851 | 15 | 15 | 7.096 | 27.71 |
| 0:NaCl - pre vs. 4.71:Cetylpyridinium - post | 2.651 | 1.325 | 1.326 | 0.1825 | 15 | 15 | 7.267 | 27.50 |
| 0:NaCl - pre vs. 6.28:NaCl - pre | 2.651 | 1.074 | 1.577 | 0.1485 | 15 | 15 | 10.62 | 14.00 |
| 0:NaCl - pre vs. 6.28:NaCl - post | 2.651 | 1.127 | 1.524 | 0.1596 | 15 | 15 | 9.548 | 14.00 |
| 0:NaCl - pre vs. 6.28:Cetylpyridinium - pre | 2.651 | 1.063 | 1.588 | 0.1807 | 15 | 15 | 8.786 | 27.33 |
| 0:NaCl - pre vs. 6.28:Cetylpyridinium - post | 2.651 | 1.097 | 1.553 | 0.1723 | 15 | 15 | 9.016 | 26.07 |
| 0:NaCl - post vs. 0:Cetylpyridinium - pre | 2.741 | 2.511 | 0.2300 | 0.2534 | 15 | 15 | 0.9077 | 24.65 |
| 0:NaCl - post vs. 0:Cetylpyridinium - post | 2.741 | 2.465 | 0.2753 | 0.2541 | 15 | 15 | 1.083 | 24.59 |
| 0:NaCl - post vs. 1.57:NaCl - pre | 2.741 | 2.051 | 0.6893 | 0.1498 | 15 | 15 | 4.603 | 14.00 |
| 0:NaCl - post vs. 1.57:NaCl - post | 2.741 | 2.138 | 0.6027 | 0.1251 | 15 | 15 | 4.817 | 14.00 |
| 0:NaCl - post vs. 1.57:Cetylpyridinium - pre | 2.741 | 2.087 | 0.6540 | 0.2215 | 15 | 15 | 2.953 | 27.18 |
| 0:NaCl - post vs. 1.57:Cetylpyridinium - post | 2.741 | 2.053 | 0.6873 | 0.2156 | 15 | 15 | 3.188 | 27.55 |
| 0:NaCl - post vs. 3.14:NaCl - pre | 2.741 | 1.679 | 1.062 | 0.1314 | 15 | 15 | 8.083 | 14.00 |
| 0:NaCl - post vs. 3.14:NaCl - post | 2.741 | 1.699 | 1.042 | 0.1330 | 15 | 15 | 7.833 | 14.00 |
| 0:NaCl - post vs. 3.14:Cetylpyridinium - pre | 2.741 | 1.704 | 1.037 | 0.2078 | 15 | 15 | 4.988 | 27.89 |
| 0:NaCl - post vs. 3.14:Cetylpyridinium - post | 2.741 | 1.608 | 1.133 | 0.2048 | 15 | 15 | 5.530 | 27.97 |
| 0:NaCl - post vs. 4.71:NaCl - pre | 2.741 | 1.351 | 1.390 | 0.1413 | 15 | 15 | 9.838 | 14.00 |
| 0:NaCl - post vs. 4.71:NaCl - post | 2.741 | 1.373 | 1.367 | 0.1582 | 15 | 15 | 8.644 | 14.00 |
| 0:NaCl - post vs. 4.71:Cetylpyridinium - pre | 2.741 | 1.337 | 1.403 | 0.1888 | 15 | 15 | 7.434 | 27.48 |
| 0:NaCl - post vs. 4.71:Cetylpyridinium - post | 2.741 | 1.325 | 1.416 | 0.1862 | 15 | 15 | 7.604 | 27.23 |
| 0:NaCl - post vs. 6.28:NaCl - pre | 2.741 | 1.074 | 1.667 | 0.1514 | 15 | 15 | 11.01 | 14.00 |
| 0:NaCl - post vs. 6.28:NaCl - post | 2.741 | 1.127 | 1.614 | 0.1659 | 15 | 15 | 9.726 | 14.00 |
| 0:NaCl - post vs. 6.28:Cetylpyridinium - pre | 2.741 | 1.063 | 1.678 | 0.1845 | 15 | 15 | 9.094 | 27.02 |
| 0:NaCl - post vs. 6.28:Cetylpyridinium - post | 2.741 | 1.097 | 1.643 | 0.1763 | 15 | 15 | 9.324 | 25.62 |
| 0:Cetylpyridinium - pre vs. 0:Cetylpyridinium - post | 2.511 | 2.465 | 0.04533 | 0.1044 | 15 | 15 | 0.4344 | 14.00 |
| 0:Cetylpyridinium - pre vs. 1.57:NaCl - pre | 2.511 | 2.051 | 0.4593 | 0.2476 | 15 | 15 | 1.855 | 23.58 |
| 0:Cetylpyridinium - pre vs. 1.57:NaCl - post | 2.511 | 2.138 | 0.3727 | 0.2477 | 15 | 15 | 1.504 | 23.60 |
| 0:Cetylpyridinium - pre vs. 1.57:Cetylpyridinium - pre | 2.511 | 2.087 | 0.4240 | 0.2091 | 15 | 15 | 2.028 | 14.00 |
| 0:Cetylpyridinium - pre vs. 1.57:Cetylpyridinium - post | 2.511 | 2.053 | 0.4573 | 0.2012 | 15 | 15 | 2.273 | 14.00 |
| 0:Cetylpyridinium - pre vs. 3.14:NaCl - pre | 2.511 | 1.679 | 0.8320 | 0.2414 | 15 | 15 | 3.447 | 22.25 |
| 0:Cetylpyridinium - pre vs. 3.14:NaCl - post | 2.511 | 1.699 | 0.8120 | 0.2352 | 15 | 15 | 3.452 | 20.80 |
| 0:Cetylpyridinium - pre vs. 3.14:Cetylpyridinium - pre | 2.511 | 1.704 | 0.8067 | 0.2016 | 15 | 15 | 4.001 | 14.00 |
| 0:Cetylpyridinium - pre vs. 3.14:Cetylpyridinium - post | 2.511 | 1.608 | 0.9027 | 0.2104 | 15 | 15 | 4.290 | 14.00 |
| 0:Cetylpyridinium - pre vs. 4.71:NaCl - pre | 2.511 | 1.351 | 1.160 | 0.2332 | 15 | 15 | 4.975 | 20.29 |
| 0:Cetylpyridinium - pre vs. 4.71:NaCl - post | 2.511 | 1.373 | 1.137 | 0.2412 | 15 | 15 | 4.716 | 22.20 |
| 0:Cetylpyridinium - pre vs. 4.71:Cetylpyridinium - pre | 2.511 | 1.337 | 1.173 | 0.1944 | 15 | 15 | 6.036 | 14.00 |
| 0:Cetylpyridinium - pre vs. 4.71:Cetylpyridinium - post | 2.511 | 1.325 | 1.186 | 0.1937 | 15 | 15 | 6.122 | 14.00 |
| 0:Cetylpyridinium - pre vs. 6.28:NaCl - pre | 2.511 | 1.074 | 1.437 | 0.2327 | 15 | 15 | 6.174 | 20.17 |
| 0:Cetylpyridinium - pre vs. 6.28:NaCl - post | 2.511 | 1.127 | 1.384 | 0.2422 | 15 | 15 | 5.715 | 22.43 |
| 0:Cetylpyridinium - pre vs. 6.28:Cetylpyridinium - pre | 2.511 | 1.063 | 1.448 | 0.1767 | 15 | 15 | 8.196 | 14.00 |
| 0:Cetylpyridinium - pre vs. 6.28:Cetylpyridinium - post | 2.511 | 1.097 | 1.413 | 0.1867 | 15 | 15 | 7.569 | 14.00 |
| 0:Cetylpyridinium - post vs. 1.57:NaCl - pre | 2.465 | 2.051 | 0.4140 | 0.2484 | 15 | 15 | 1.667 | 23.52 |
| 0:Cetylpyridinium - post vs. 1.57:NaCl - post | 2.465 | 2.138 | 0.3273 | 0.2485 | 15 | 15 | 1.317 | 23.54 |
| 0:Cetylpyridinium - post vs. 1.57:Cetylpyridinium - pre | 2.465 | 2.087 | 0.3787 | 0.1602 | 15 | 15 | 2.364 | 14.00 |
| 0:Cetylpyridinium - post vs. 1.57:Cetylpyridinium - post | 2.465 | 2.053 | 0.4120 | 0.1551 | 15 | 15 | 2.657 | 14.00 |
| 0:Cetylpyridinium - post vs. 3.14:NaCl - pre | 2.465 | 1.679 | 0.7867 | 0.2422 | 15 | 15 | 3.248 | 22.20 |
| 0:Cetylpyridinium - post vs. 3.14:NaCl - post | 2.465 | 1.699 | 0.7667 | 0.2360 | 15 | 15 | 3.248 | 20.75 |
| 0:Cetylpyridinium - post vs. 3.14:Cetylpyridinium - pre | 2.465 | 1.704 | 0.7613 | 0.1622 | 15 | 15 | 4.694 | 14.00 |
| 0:Cetylpyridinium - post vs. 3.14:Cetylpyridinium - post | 2.465 | 1.608 | 0.8573 | 0.1683 | 15 | 15 | 5.094 | 14.00 |
| 0:Cetylpyridinium - post vs. 4.71:NaCl - pre | 2.465 | 1.351 | 1.115 | 0.2340 | 15 | 15 | 4.764 | 20.25 |
| 0:Cetylpyridinium - post vs. 4.71:NaCl - post | 2.465 | 1.373 | 1.092 | 0.2419 | 15 | 15 | 4.513 | 22.14 |
| 0:Cetylpyridinium - post vs. 4.71:Cetylpyridinium - pre | 2.465 | 1.337 | 1.128 | 0.1610 | 15 | 15 | 7.006 | 14.00 |
| 0:Cetylpyridinium - post vs. 4.71:Cetylpyridinium - post | 2.465 | 1.325 | 1.141 | 0.1608 | 15 | 15 | 7.093 | 14.00 |
| 0:Cetylpyridinium - post vs. 6.28:NaCl - pre | 2.465 | 1.074 | 1.391 | 0.2335 | 15 | 15 | 5.958 | 20.12 |
| 0:Cetylpyridinium - post vs. 6.28:NaCl - post | 2.465 | 1.127 | 1.339 | 0.2430 | 15 | 15 | 5.510 | 22.37 |
| 0:Cetylpyridinium - post vs. 6.28:Cetylpyridinium - pre | 2.465 | 1.063 | 1.403 | 0.1499 | 15 | 15 | 9.360 | 14.00 |
| 0:Cetylpyridinium - post vs. 6.28:Cetylpyridinium - post | 2.465 | 1.097 | 1.368 | 0.1577 | 15 | 15 | 8.672 | 14.00 |
| 1.57:NaCl - pre vs. 1.57:NaCl - post | 2.051 | 2.138 | -0.08667 | 0.06288 | 15 | 15 | 1.378 | 14.00 |
| 1.57:NaCl - pre vs. 1.57:Cetylpyridinium - pre | 2.051 | 2.087 | -0.03533 | 0.2148 | 15 | 15 | 0.1645 | 26.39 |
| 1.57:NaCl - pre vs. 1.57:Cetylpyridinium - post | 2.051 | 2.053 | -0.002000 | 0.2088 | 15 | 15 | 0.009578 | 26.89 |
| 1.57:NaCl - pre vs. 3.14:NaCl - pre | 2.051 | 1.679 | 0.3727 | 0.05504 | 15 | 15 | 6.771 | 14.00 |
| 1.57:NaCl - pre vs. 3.14:NaCl - post | 2.051 | 1.699 | 0.3527 | 0.05910 | 15 | 15 | 5.967 | 14.00 |
| 1.57:NaCl - pre vs. 3.14:Cetylpyridinium - pre | 2.051 | 1.704 | 0.3473 | 0.2008 | 15 | 15 | 1.730 | 27.48 |
| 1.57:NaCl - pre vs. 3.14:Cetylpyridinium - post | 2.051 | 1.608 | 0.4433 | 0.1977 | 15 | 15 | 2.243 | 27.66 |
| 1.57:NaCl - pre vs. 4.71:NaCl - pre | 2.051 | 1.351 | 0.7007 | 0.06562 | 15 | 15 | 10.68 | 14.00 |
| 1.57:NaCl - pre vs. 4.71:NaCl - post | 2.051 | 1.373 | 0.6780 | 0.07351 | 15 | 15 | 9.223 | 14.00 |
| 1.57:NaCl - pre vs. 4.71:Cetylpyridinium - pre | 2.051 | 1.337 | 0.7140 | 0.1810 | 15 | 15 | 3.945 | 27.90 |
| 1.57:NaCl - pre vs. 4.71:Cetylpyridinium - post | 2.051 | 1.325 | 0.7267 | 0.1783 | 15 | 15 | 4.076 | 27.76 |
| 1.57:NaCl - pre vs. 6.28:NaCl - pre | 2.051 | 1.074 | 0.9773 | 0.08706 | 15 | 15 | 11.23 | 14.00 |
| 1.57:NaCl - pre vs. 6.28:NaCl - post | 2.051 | 1.127 | 0.9247 | 0.08147 | 15 | 15 | 11.35 | 14.00 |
| 1.57:NaCl - pre vs. 6.28:Cetylpyridinium - pre | 2.051 | 1.063 | 0.9887 | 0.1765 | 15 | 15 | 5.601 | 27.63 |
| 1.57:NaCl - pre vs. 6.28:Cetylpyridinium - post | 2.051 | 1.097 | 0.9540 | 0.1679 | 15 | 15 | 5.683 | 26.55 |
| 1.57:NaCl - post vs. 1.57:Cetylpyridinium - pre | 2.138 | 2.087 | 0.05133 | 0.2150 | 15 | 15 | 0.2388 | 26.41 |
| 1.57:NaCl - post vs. 1.57:Cetylpyridinium - post | 2.138 | 2.053 | 0.08467 | 0.2089 | 15 | 15 | 0.4053 | 26.91 |
| 1.57:NaCl - post vs. 3.14:NaCl - pre | 2.138 | 1.679 | 0.4593 | 0.06248 | 15 | 15 | 7.351 | 14.00 |
| 1.57:NaCl - post vs. 3.14:NaCl - post | 2.138 | 1.699 | 0.4393 | 0.03866 | 15 | 15 | 11.36 | 14.00 |
| 1.57:NaCl - post vs. 3.14:Cetylpyridinium - pre | 2.138 | 1.704 | 0.4340 | 0.2009 | 15 | 15 | 2.161 | 27.49 |
| 1.57:NaCl - post vs. 3.14:Cetylpyridinium - post | 2.138 | 1.608 | 0.5300 | 0.1978 | 15 | 15 | 2.680 | 27.67 |
| 1.57:NaCl - post vs. 4.71:NaCl - pre | 2.138 | 1.351 | 0.7873 | 0.07656 | 15 | 15 | 10.28 | 14.00 |
| 1.57:NaCl - post vs. 4.71:NaCl - post | 2.138 | 1.373 | 0.7647 | 0.07227 | 15 | 15 | 10.58 | 14.00 |
| 1.57:NaCl - post vs. 4.71:Cetylpyridinium - pre | 2.138 | 1.337 | 0.8007 | 0.1811 | 15 | 15 | 4.421 | 27.89 |
| 1.57:NaCl - post vs. 4.71:Cetylpyridinium - post | 2.138 | 1.325 | 0.8133 | 0.1784 | 15 | 15 | 4.558 | 27.75 |
| 1.57:NaCl - post vs. 6.28:NaCl - pre | 2.138 | 1.074 | 1.064 | 0.09678 | 15 | 15 | 10.99 | 14.00 |
| 1.57:NaCl - post vs. 6.28:NaCl - post | 2.138 | 1.127 | 1.011 | 0.08912 | 15 | 15 | 11.35 | 14.00 |
| 1.57:NaCl - post vs. 6.28:Cetylpyridinium - pre | 2.138 | 1.063 | 1.075 | 0.1767 | 15 | 15 | 6.087 | 27.62 |
| 1.57:NaCl - post vs. 6.28:Cetylpyridinium - post | 2.138 | 1.097 | 1.041 | 0.1680 | 15 | 15 | 6.194 | 26.54 |
| 1.57:Cetylpyridinium - pre vs. 1.57:Cetylpyridinium - post | 2.087 | 2.053 | 0.03333 | 0.05639 | 15 | 15 | 0.5911 | 14.00 |
| 1.57:Cetylpyridinium - pre vs. 3.14:NaCl - pre | 2.087 | 1.679 | 0.4080 | 0.2076 | 15 | 15 | 1.965 | 25.17 |
| 1.57:Cetylpyridinium - pre vs. 3.14:NaCl - post | 2.087 | 1.699 | 0.3880 | 0.2004 | 15 | 15 | 1.936 | 23.58 |
| 1.57:Cetylpyridinium - pre vs. 3.14:Cetylpyridinium - pre | 2.087 | 1.704 | 0.3827 | 0.04180 | 15 | 15 | 9.155 | 14.00 |
| 1.57:Cetylpyridinium - pre vs. 3.14:Cetylpyridinium - post | 2.087 | 1.608 | 0.4787 | 0.05994 | 15 | 15 | 7.985 | 14.00 |
| 1.57:Cetylpyridinium - pre vs. 4.71:NaCl - pre | 2.087 | 1.351 | 0.7360 | 0.1980 | 15 | 15 | 3.717 | 22.97 |
| 1.57:Cetylpyridinium - pre vs. 4.71:NaCl - post | 2.087 | 1.373 | 0.7133 | 0.2074 | 15 | 15 | 3.440 | 25.12 |
| 1.57:Cetylpyridinium - pre vs. 4.71:Cetylpyridinium - pre | 2.087 | 1.337 | 0.7493 | 0.06323 | 15 | 15 | 11.85 | 14.00 |
| 1.57:Cetylpyridinium - pre vs. 4.71:Cetylpyridinium - post | 2.087 | 1.325 | 0.7620 | 0.07185 | 15 | 15 | 10.61 | 14.00 |
| 1.57:Cetylpyridinium - pre vs. 6.28:NaCl - pre | 2.087 | 1.074 | 1.013 | 0.1974 | 15 | 15 | 5.129 | 22.82 |
| 1.57:Cetylpyridinium - pre vs. 6.28:NaCl - post | 2.087 | 1.127 | 0.9600 | 0.2085 | 15 | 15 | 4.603 | 25.35 |
| 1.57:Cetylpyridinium - pre vs. 6.28:Cetylpyridinium - pre | 2.087 | 1.063 | 1.024 | 0.1003 | 15 | 15 | 10.21 | 14.00 |
| 1.57:Cetylpyridinium - pre vs. 6.28:Cetylpyridinium - post | 2.087 | 1.097 | 0.9893 | 0.09432 | 15 | 15 | 10.49 | 14.00 |
| 1.57:Cetylpyridinium - post vs. 3.14:NaCl - pre | 2.053 | 1.679 | 0.3747 | 0.2014 | 15 | 15 | 1.861 | 25.78 |
| 1.57:Cetylpyridinium - post vs. 3.14:NaCl - post | 2.053 | 1.699 | 0.3547 | 0.1939 | 15 | 15 | 1.829 | 24.23 |
| 1.57:Cetylpyridinium - post vs. 3.14:Cetylpyridinium - pre | 2.053 | 1.704 | 0.3493 | 0.06092 | 15 | 15 | 5.735 | 14.00 |
| 1.57:Cetylpyridinium - post vs. 3.14:Cetylpyridinium - post | 2.053 | 1.608 | 0.4453 | 0.04256 | 15 | 15 | 10.46 | 14.00 |
| 1.57:Cetylpyridinium - post vs. 4.71:NaCl - pre | 2.053 | 1.351 | 0.7027 | 0.1915 | 15 | 15 | 3.670 | 23.62 |
| 1.57:Cetylpyridinium - post vs. 4.71:NaCl - post | 2.053 | 1.373 | 0.6800 | 0.2011 | 15 | 15 | 3.382 | 25.73 |
| 1.57:Cetylpyridinium - post vs. 4.71:Cetylpyridinium - pre | 2.053 | 1.337 | 0.7160 | 0.07548 | 15 | 15 | 9.486 | 14.00 |
| 1.57:Cetylpyridinium - post vs. 4.71:Cetylpyridinium - post | 2.053 | 1.325 | 0.7287 | 0.06549 | 15 | 15 | 11.13 | 14.00 |
| 1.57:Cetylpyridinium - post vs. 6.28:NaCl - pre | 2.053 | 1.074 | 0.9793 | 0.1909 | 15 | 15 | 5.131 | 23.46 |
| 1.57:Cetylpyridinium - post vs. 6.28:NaCl - post | 2.053 | 1.127 | 0.9267 | 0.2023 | 15 | 15 | 4.580 | 25.95 |
| 1.57:Cetylpyridinium - post vs. 6.28:Cetylpyridinium - pre | 2.053 | 1.063 | 0.9907 | 0.1144 | 15 | 15 | 8.659 | 14.00 |
| 1.57:Cetylpyridinium - post vs. 6.28:Cetylpyridinium - post | 2.053 | 1.097 | 0.9560 | 0.09017 | 15 | 15 | 10.60 | 14.00 |
| 3.14:NaCl - pre vs. 3.14:NaCl - post | 1.679 | 1.699 | -0.02000 | 0.04924 | 15 | 15 | 0.4062 | 14.00 |
| 3.14:NaCl - pre vs. 3.14:Cetylpyridinium - pre | 1.679 | 1.704 | -0.02533 | 0.1930 | 15 | 15 | 0.1313 | 26.59 |
| 3.14:NaCl - pre vs. 3.14:Cetylpyridinium - post | 1.679 | 1.608 | 0.07067 | 0.1898 | 15 | 15 | 0.3724 | 26.88 |
| 3.14:NaCl - pre vs. 4.71:NaCl - pre | 1.679 | 1.351 | 0.3280 | 0.04244 | 15 | 15 | 7.729 | 14.00 |
| 3.14:NaCl - pre vs. 4.71:NaCl - post | 1.679 | 1.373 | 0.3053 | 0.07618 | 15 | 15 | 4.008 | 14.00 |
| 3.14:NaCl - pre vs. 4.71:Cetylpyridinium - pre | 1.679 | 1.337 | 0.3413 | 0.1723 | 15 | 15 | 1.981 | 27.97 |
| 3.14:NaCl - pre vs. 4.71:Cetylpyridinium - post | 1.679 | 1.325 | 0.3540 | 0.1695 | 15 | 15 | 2.088 | 28.00 |
| 3.14:NaCl - pre vs. 6.28:NaCl - pre | 1.679 | 1.074 | 0.6047 | 0.07807 | 15 | 15 | 7.746 | 14.00 |
| 3.14:NaCl - pre vs. 6.28:NaCl - post | 1.679 | 1.127 | 0.5520 | 0.08069 | 15 | 15 | 6.841 | 14.00 |
| 3.14:NaCl - pre vs. 6.28:Cetylpyridinium - pre | 1.679 | 1.063 | 0.6160 | 0.1677 | 15 | 15 | 3.674 | 27.99 |
| 3.14:NaCl - pre vs. 6.28:Cetylpyridinium - post | 1.679 | 1.097 | 0.5813 | 0.1585 | 15 | 15 | 3.667 | 27.46 |
| 3.14:NaCl - post vs. 3.14:Cetylpyridinium - pre | 1.699 | 1.704 | -0.005333 | 0.1852 | 15 | 15 | 0.02879 | 25.16 |
| 3.14:NaCl - post vs. 3.14:Cetylpyridinium - post | 1.699 | 1.608 | 0.09067 | 0.1819 | 15 | 15 | 0.4985 | 25.52 |
| 3.14:NaCl - post vs. 4.71:NaCl - pre | 1.699 | 1.351 | 0.3480 | 0.04643 | 15 | 15 | 7.496 | 14.00 |
| 3.14:NaCl - post vs. 4.71:NaCl - post | 1.699 | 1.373 | 0.3253 | 0.04654 | 15 | 15 | 6.991 | 14.00 |
| 3.14:NaCl - post vs. 4.71:Cetylpyridinium - pre | 1.699 | 1.337 | 0.3613 | 0.1636 | 15 | 15 | 2.209 | 27.39 |
| 3.14:NaCl - post vs. 4.71:Cetylpyridinium - post | 1.699 | 1.325 | 0.3740 | 0.1606 | 15 | 15 | 2.328 | 27.62 |
| 3.14:NaCl - post vs. 6.28:NaCl - pre | 1.699 | 1.074 | 0.6247 | 0.06607 | 15 | 15 | 9.455 | 14.00 |
| 3.14:NaCl - post vs. 6.28:NaCl - post | 1.699 | 1.127 | 0.5720 | 0.06075 | 15 | 15 | 9.416 | 14.00 |
| 3.14:NaCl - post vs. 6.28:Cetylpyridinium - pre | 1.699 | 1.063 | 0.6360 | 0.1587 | 15 | 15 | 4.009 | 27.75 |
| 3.14:NaCl - post vs. 6.28:Cetylpyridinium - post | 1.699 | 1.097 | 0.6013 | 0.1490 | 15 | 15 | 4.037 | 27.98 |
| 3.14:Cetylpyridinium - pre vs. 3.14:Cetylpyridinium - post | 1.704 | 1.608 | 0.09600 | 0.04737 | 15 | 15 | 2.027 | 14.00 |
| 3.14:Cetylpyridinium - pre vs. 4.71:NaCl - pre | 1.704 | 1.351 | 0.3533 | 0.1826 | 15 | 15 | 1.935 | 24.56 |
| 3.14:Cetylpyridinium - pre vs. 4.71:NaCl - post | 1.704 | 1.373 | 0.3307 | 0.1927 | 15 | 15 | 1.716 | 26.54 |
| 3.14:Cetylpyridinium - pre vs. 4.71:Cetylpyridinium - pre | 1.704 | 1.337 | 0.3667 | 0.03819 | 15 | 15 | 9.600 | 14.00 |
| 3.14:Cetylpyridinium - pre vs. 4.71:Cetylpyridinium - post | 1.704 | 1.325 | 0.3793 | 0.05229 | 15 | 15 | 7.254 | 14.00 |
| 3.14:Cetylpyridinium - pre vs. 6.28:NaCl - pre | 1.704 | 1.074 | 0.6300 | 0.1820 | 15 | 15 | 3.461 | 24.40 |
| 3.14:Cetylpyridinium - pre vs. 6.28:NaCl - post | 1.704 | 1.127 | 0.5773 | 0.1940 | 15 | 15 | 2.976 | 26.73 |
| 3.14:Cetylpyridinium - pre vs. 6.28:Cetylpyridinium - pre | 1.704 | 1.063 | 0.6413 | 0.08378 | 15 | 15 | 7.655 | 14.00 |
| 3.14:Cetylpyridinium - pre vs. 6.28:Cetylpyridinium - post | 1.704 | 1.097 | 0.6067 | 0.07541 | 15 | 15 | 8.045 | 14.00 |
| 3.14:Cetylpyridinium - post vs. 4.71:NaCl - pre | 1.608 | 1.351 | 0.2573 | 0.1792 | 15 | 15 | 1.436 | 24.94 |
| 3.14:Cetylpyridinium - post vs. 4.71:NaCl - post | 1.608 | 1.373 | 0.2347 | 0.1895 | 15 | 15 | 1.238 | 26.84 |
| 3.14:Cetylpyridinium - post vs. 4.71:Cetylpyridinium - pre | 1.608 | 1.337 | 0.2707 | 0.05220 | 15 | 15 | 5.185 | 14.00 |
| 3.14:Cetylpyridinium - post vs. 4.71:Cetylpyridinium - post | 1.608 | 1.325 | 0.2833 | 0.03734 | 15 | 15 | 7.589 | 14.00 |
| 3.14:Cetylpyridinium - post vs. 6.28:NaCl - pre | 1.608 | 1.074 | 0.5340 | 0.1786 | 15 | 15 | 2.990 | 24.79 |
| 3.14:Cetylpyridinium - post vs. 6.28:NaCl - post | 1.608 | 1.127 | 0.4813 | 0.1908 | 15 | 15 | 2.523 | 27.01 |
| 3.14:Cetylpyridinium - post vs. 6.28:Cetylpyridinium - pre | 1.608 | 1.063 | 0.5453 | 0.09867 | 15 | 15 | 5.527 | 14.00 |
| 3.14:Cetylpyridinium - post vs. 6.28:Cetylpyridinium - post | 1.608 | 1.097 | 0.5107 | 0.06796 | 15 | 15 | 7.514 | 14.00 |
| 4.71:NaCl - pre vs. 4.71:NaCl - post | 1.351 | 1.373 | -0.02267 | 0.05578 | 15 | 15 | 0.4064 | 14.00 |
| 4.71:NaCl - pre vs. 4.71:Cetylpyridinium - pre | 1.351 | 1.337 | 0.01333 | 0.1606 | 15 | 15 | 0.08300 | 27.01 |
| 4.71:NaCl - pre vs. 4.71:Cetylpyridinium - post | 1.351 | 1.325 | 0.02600 | 0.1576 | 15 | 15 | 0.1649 | 27.30 |
| 4.71:NaCl - pre vs. 6.28:NaCl - pre | 1.351 | 1.074 | 0.2767 | 0.04464 | 15 | 15 | 6.197 | 14.00 |
| 4.71:NaCl - pre vs. 6.28:NaCl - post | 1.351 | 1.127 | 0.2240 | 0.05317 | 15 | 15 | 4.213 | 14.00 |
| 4.71:NaCl - pre vs. 6.28:Cetylpyridinium - pre | 1.351 | 1.063 | 0.2880 | 0.1556 | 15 | 15 | 1.851 | 27.48 |
| 4.71:NaCl - pre vs. 6.28:Cetylpyridinium - post | 1.351 | 1.097 | 0.2533 | 0.1457 | 15 | 15 | 1.738 | 27.99 |
| 4.71:NaCl - post vs. 4.71:Cetylpyridinium - pre | 1.373 | 1.337 | 0.03600 | 0.1720 | 15 | 15 | 0.2093 | 27.96 |
| 4.71:NaCl - post vs. 4.71:Cetylpyridinium - post | 1.373 | 1.325 | 0.04867 | 0.1692 | 15 | 15 | 0.2876 | 28.00 |
| 4.71:NaCl - post vs. 6.28:NaCl - pre | 1.373 | 1.074 | 0.2993 | 0.04898 | 15 | 15 | 6.111 | 14.00 |
| 4.71:NaCl - post vs. 6.28:NaCl - post | 1.373 | 1.127 | 0.2467 | 0.02552 | 15 | 15 | 9.667 | 14.00 |
| 4.71:NaCl - post vs. 6.28:Cetylpyridinium - pre | 1.373 | 1.063 | 0.3107 | 0.1673 | 15 | 15 | 1.857 | 27.99 |
| 4.71:NaCl - post vs. 6.28:Cetylpyridinium - post | 1.373 | 1.097 | 0.2760 | 0.1582 | 15 | 15 | 1.745 | 27.49 |
| 4.71:Cetylpyridinium - pre vs. 4.71:Cetylpyridinium - post | 1.337 | 1.325 | 0.01267 | 0.03631 | 15 | 15 | 0.3488 | 14.00 |
| 4.71:Cetylpyridinium - pre vs. 6.28:NaCl - pre | 1.337 | 1.074 | 0.2633 | 0.1599 | 15 | 15 | 1.647 | 26.90 |
| 4.71:Cetylpyridinium - pre vs. 6.28:NaCl - post | 1.337 | 1.127 | 0.2107 | 0.1734 | 15 | 15 | 1.215 | 27.99 |
| 4.71:Cetylpyridinium - pre vs. 6.28:Cetylpyridinium - pre | 1.337 | 1.063 | 0.2747 | 0.06159 | 15 | 15 | 4.460 | 14.00 |
| 4.71:Cetylpyridinium - pre vs. 6.28:Cetylpyridinium - post | 1.337 | 1.097 | 0.2400 | 0.05378 | 15 | 15 | 4.463 | 14.00 |
| 4.71:Cetylpyridinium - post vs. 6.28:NaCl - pre | 1.325 | 1.074 | 0.2507 | 0.1569 | 15 | 15 | 1.598 | 27.21 |
| 4.71:Cetylpyridinium - post vs. 6.28:NaCl - post | 1.325 | 1.127 | 0.1980 | 0.1707 | 15 | 15 | 1.160 | 28.00 |
| 4.71:Cetylpyridinium - post vs. 6.28:Cetylpyridinium - pre | 1.325 | 1.063 | 0.2620 | 0.06958 | 15 | 15 | 3.766 | 14.00 |
| 4.71:Cetylpyridinium - post vs. 6.28:Cetylpyridinium - post | 1.325 | 1.097 | 0.2273 | 0.03687 | 15 | 15 | 6.166 | 14.00 |
| 6.28:NaCl - pre vs. 6.28:NaCl - post | 1.074 | 1.127 | -0.05267 | 0.04131 | 15 | 15 | 1.275 | 14.00 |
| 6.28:NaCl - pre vs. 6.28:Cetylpyridinium - pre | 1.074 | 1.063 | 0.01133 | 0.1549 | 15 | 15 | 0.07318 | 27.39 |
| 6.28:NaCl - pre vs. 6.28:Cetylpyridinium - post | 1.074 | 1.097 | -0.02333 | 0.1449 | 15 | 15 | 0.1610 | 27.98 |
| 6.28:NaCl - post vs. 6.28:Cetylpyridinium - pre | 1.127 | 1.063 | 0.06400 | 0.1688 | 15 | 15 | 0.3791 | 27.97 |
| 6.28:NaCl - post vs. 6.28:Cetylpyridinium - post | 1.127 | 1.097 | 0.02933 | 0.1597 | 15 | 15 | 0.1836 | 27.36 |
| 6.28:Cetylpyridinium - pre vs. 6.28:Cetylpyridinium - post | 1.063 | 1.097 | -0.03467 | 0.05449 | 15 | 15 | 0.6362 | 14.00 |

| Table Analyzed | Pmax |  |  |  |  |
| --- | --- | --- | --- | --- | --- |
|  |  |  |  |  |  |
| Two-way RM ANOVA | Matching: Across row |  |  |  |  |
| Assume sphericity? | Yes |  |  |  |  |
| Alpha | 0.05 |  |  |  |  |
|  |  |  |  |  |  |
| Source of Variation | % of total variation | P value | P value summary | Significant? |  |
| Time x Treatment | 0.1649 | 0.3288 | ns | No |  |
| Time | 0.1176 | 0.8536 | ns | No |  |
| Treatment | 0.1022 | 0.4405 | ns | No |  |
| Subject | 94.94 | <0.0001 | **** | Yes |  |
|  |  |  |  |  |  |
| ANOVA table | SS | DF | MS | F (DFn, DFd) | P value |
| Time x Treatment | 0.6615 | 1 | 0.6615 | F (1, 28) = 0.9878 | P=0.3288 |
| Time | 0.4717 | 1 | 0.4717 | F (1, 28) = 0.03467 | P=0.8536 |
| Treatment | 0.4100 | 1 | 0.4100 | F (1, 28) = 0.6123 | P=0.4405 |
| Subject | 380.9 | 28 | 13.60 | F (28, 28) = 20.31 | P<0.0001 |
| Residual | 18.75 | 28 | 0.6697 |  |  |
|  |  |  |  |  |  |
| Difference between row means |  |  |  |  |  |
| Mean of NaCl | 7.320 |  |  |  |  |
| Mean of Cetylpyridinium | 7.143 |  |  |  |  |
| Difference between means | 0.1773 |  |  |  |  |
| SE of difference | 0.9523 |  |  |  |  |
| 95% CI of difference | -1.773 to 2.128 |  |  |  |  |
|  |  |  |  |  |  |
| Difference between column means |  |  |  |  |  |
| Mean of Pre | 7.149 |  |  |  |  |
| Mean of Post | 7.314 |  |  |  |  |
| Difference between means | -0.1653 |  |  |  |  |
| SE of difference | 0.2113 |  |  |  |  |
| 95% CI of difference | -0.5981 to 0.2675 |  |  |  |  |
|  |  |  |  |  |  |
| Interaction CI |  |  |  |  |  |
| Mean diff, A1 - B1 | -0.3753 |  |  |  |  |
| Mean diff, A2 - B2 | 0.04467 |  |  |  |  |
| (A1 -B1) - (A2 - B2) | -0.4200 |  |  |  |  |
| 95% CI of difference | -1.286 to 0.4456 |  |  |  |  |
| (B1 - A1) - (B2 - A2) | 0.4200 |  |  |  |  |
| 95% CI of difference | -0.4456 to 1.286 |  |  |  |  |
|  |  |  |  |  |  |
| Data summary |  |  |  |  |  |
| Number of columns (Treatment) | 2 |  |  |  |  |
| Number of rows (Time) | 2 |  |  |  |  |
| Number of subjects (Subject) | 30 |  |  |  |  |
| Number of missing values | 0 |  |  |  |  |

| Compare each cell mean with the other cell mean in that row |  |  |  |  |  |  |  |  |
| --- | --- | --- | --- | --- | --- | --- | --- | --- |
|  |  |  |  |  |  |  |  |  |
| Number of families | 1 |  |  |  |  |  |  |  |
| Number of comparisons per family | 2 |  |  |  |  |  |  |  |
| Alpha | 0.05 |  |  |  |  |  |  |  |
|  |  |  |  |  |  |  |  |  |
| Holm-Šídák's multiple comparisons test | Mean Diff. | Below threshold? | Summary | Adjusted P Value |  |  |  |  |
|  |  |  |  |  |  |  |  |  |
| Pre - Post |  |  |  |  |  |  |  |  |
| NaCl | -0.3753 | No | ns | 0.3908 |  |  |  |  |
| Cetylpyridinium | 0.04467 | No | ns | 0.8822 |  |  |  |  |
|  |  |  |  |  |  |  |  |  |
|  |  |  |  |  |  |  |  |  |
| Test details | Mean 1 | Mean 2 | Mean Diff. | SE of diff. | N1 | N2 | t | DF |
|  |  |  |  |  |  |  |  |  |
| Pre - Post |  |  |  |  |  |  |  |  |
| NaCl | 7.133 | 7.508 | -0.3753 | 0.2988 | 15 | 15 | 1.256 | 28.00 |
| Cetylpyridinium | 7.165 | 7.121 | 0.04467 | 0.2988 | 15 | 15 | 0.1495 | 28.00 |

| Table Analyzed | Vmax |  |  |  |  |
| --- | --- | --- | --- | --- | --- |
|  |  |  |  |  |  |
| Two-way RM ANOVA | Matching: Across row |  |  |  |  |
| Assume sphericity? | Yes |  |  |  |  |
| Alpha | 0.05 |  |  |  |  |
|  |  |  |  |  |  |
| Source of Variation | % of total variation | P value | P value summary | Significant? |  |
| Time x Treatment | 0.2892 | 0.4875 | ns | No |  |
| Time | 0.002195 | 0.9784 | ns | No |  |
| Treatment | 1.203 | 0.1624 | ns | No |  |
| Subject | 82.15 | <0.0001 | **** | Yes |  |
|  |  |  |  |  |  |
| ANOVA table | SS | DF | MS | F (DFn, DFd) | P value |
| Time x Treatment | 1.700 | 1 | 1.700 | F (1, 28) = 0.4950 | P=0.4875 |
| Time | 0.01291 | 1 | 0.01291 | F (1, 28) = 0.0007483 | P=0.9784 |
| Treatment | 7.073 | 1 | 7.073 | F (1, 28) = 2.059 | P=0.1624 |
| Subject | 483.0 | 28 | 17.25 | F (28, 28) = 5.022 | P<0.0001 |
| Residual | 96.17 | 28 | 3.435 |  |  |
|  |  |  |  |  |  |
| Difference between row means |  |  |  |  |  |
| Mean of NaCl | 12.88 |  |  |  |  |
| Mean of Cetylpyridinium | 12.85 |  |  |  |  |
| Difference between means | 0.02933 |  |  |  |  |
| SE of difference | 1.072 |  |  |  |  |
| 95% CI of difference | -2.167 to 2.226 |  |  |  |  |
|  |  |  |  |  |  |
| Difference between column means |  |  |  |  |  |
| Mean of Pre | 12.52 |  |  |  |  |
| Mean of Post | 13.21 |  |  |  |  |
| Difference between means | -0.6867 |  |  |  |  |
| SE of difference | 0.4785 |  |  |  |  |
| 95% CI of difference | -1.667 to 0.2935 |  |  |  |  |
|  |  |  |  |  |  |
| Interaction CI |  |  |  |  |  |
| Mean diff, A1 - B1 | -0.3500 |  |  |  |  |
| Mean diff, A2 - B2 | -1.023 |  |  |  |  |
| (A1 -B1) - (A2 - B2) | 0.6733 |  |  |  |  |
| 95% CI of difference | -1.287 to 2.634 |  |  |  |  |
| (B1 - A1) - (B2 - A2) | -0.6733 |  |  |  |  |
| 95% CI of difference | -2.634 to 1.287 |  |  |  |  |
|  |  |  |  |  |  |
| Data summary |  |  |  |  |  |
| Number of columns (Treatment) | 2 |  |  |  |  |
| Number of rows (Time) | 2 |  |  |  |  |
| Number of subjects (Subject) | 30 |  |  |  |  |
| Number of missing values | 0 |  |  |  |  |

| Compare each cell mean with the other cell mean in that row |  |  |  |  |  |  |  |  |
| --- | --- | --- | --- | --- | --- | --- | --- | --- |
|  |  |  |  |  |  |  |  |  |
| Number of families | 1 |  |  |  |  |  |  |  |
| Number of comparisons per family | 2 |  |  |  |  |  |  |  |
| Alpha | 0.05 |  |  |  |  |  |  |  |
|  |  |  |  |  |  |  |  |  |
| Holm-Šídák's multiple comparisons test | Mean Diff. | Below threshold? | Summary | Adjusted P Value |  |  |  |  |
|  |  |  |  |  |  |  |  |  |
| Pre - Post |  |  |  |  |  |  |  |  |
| NaCl | -0.3500 | No | ns | 0.6091 |  |  |  |  |
| Cetylpyridinium | -1.023 | No | ns | 0.2633 |  |  |  |  |
|  |  |  |  |  |  |  |  |  |
|  |  |  |  |  |  |  |  |  |
| Test details | Mean 1 | Mean 2 | Mean Diff. | SE of diff. | N1 | N2 | t | DF |
|  |  |  |  |  |  |  |  |  |
| Pre - Post |  |  |  |  |  |  |  |  |
| NaCl | 12.70 | 13.05 | -0.3500 | 0.6767 | 15 | 15 | 0.5172 | 28.00 |
| Cetylpyridinium | 12.34 | 13.36 | -1.023 | 0.6767 | 15 | 15 | 1.512 | 28.00 |
