# Supplementary material for: Pilot randomized trial of the effect of antibacterial mouthwash on muscle contractile function in healthy young adults
Source: PLoS One. 2025 Feb 12;20(2):e0312961. doi: 10.1371/journal.pone.0312961 (PMC11819566; doi:10.1371/journal.pone.0312961)
Supplement: Attachment — Submitted filename: Response to Reviewers.docx [file pone.0312961.s004.docx]

Reviewer #1: General Comments: The authors have provided a well written manuscript focused on investigating the effect of mouthwash on oral biomarkers of nitric oxide bioavailability and lower extremity skeletal muscle function. Although the question is of relevance given the increasing interest in nitrate supplementation, the population for testing their hypothesis is questionable. Given the support provided in the introduction and the exclusionary criteria of several of the author's manuscripts, it would seem that young healthy adults would not benefit from nitrate supplementation, possibly due to the robustness of physiological reserves at that developmental stage. Thus, the blunting of the enterosalivary pathway with mouthwash would need to have large effect to even expect a reduction in skeletal muscle function in this population. As the authors allude in the discussion, studying this effect in older adults or patient populations that have documented poor NO bioavailability would seem to be the ideal population, especially given emerging evidence of links between oral health and frailty. Nonetheless, in accordance with PLOS ONE publication criteria, the study was conducted well, and I only have minor edits about the framing of the results, questionable statistical analyses, and the preliminary nature of the study.

**We thank the reviewer for their comments on our manuscript. However, we disagree with their assertion that we tested our hypothesis in the wrong population.**

**First, although we have published a number of studies of dietary nitrate supplementation in older men and women and patients with heart failure, we have also previously reported that this intervention increases neuromuscular function in healthy young and middle aged untrained men and women (PMCID: PMC4362985) as well as in young athletes (PMCID: PMC4889556). In a follow-up study, we found no apparent effect of age on the magnitude of this effect (PMCID: PMC5789728). This conclusion was supported by our later independent-participant data meta-analysis of the literature (PMCID: PMC8501726), in which the vast majority of participants (i.e., 230 out of 268, or 86%) were young and healthy. It is therefore clear that, contrary to the reviewer’s assertion, dietary nitrate supplementation is beneficial even in individuals such as those included in the present study.**

**Second, and more importantly, the purpose of the present study was actually to examine the converse question, i.e., to determine whether over-the-counter mouthwash reduced NO levels, and if so, whether this would diminish muscle function. Logically, those most likely to be impacted would be those with normal/adequate levels in the first place – those in whom NO production is already reduced (e.g., older individuals) would presumably be *less* likely to be impacted (“floor effect”). As it turned out, cetylpyrdinium-based mouthwash seemed to have only a slight influence on salivary markers of NO bioavailability, but this could not have been predicted *a priori*, as no previous study had tested the effects of cetylpyridinium-containing mouthwash in isolation, only in combination with nitrate supplementation (Ref. 34).**

Specific:

Title Page: This should be labelled a pilot randomized trial for several reasons: 1) No sample size calculation was performed and the effect size from the meta-analysis includes older adults, who we would expect to have a large effect; 2) only 13/15 provided saliva, no blood NO biomarkers, and only 8/15 provided breath NO; 3) The one significant result is potentially driven by an outlier (well discuss further in methods).

**Per the reviewer’s suggestion, the title of the manuscript has been changed to “Pilot randomized trial of the effect of antibacterial mouthwash on muscle contractile function in healthy young adults”**

Abstract: The authors state "Cetylpyridinium mouthwash INHIBITS reduction of nitrate to nitrite...". This was not tested in this study. The mouthwash was associated with a reduction in salivary NO precursors. Please edit accordingly.

**The sentence in question has been changed to read “Cetylpyridinium mouthwash reduces the relative abundance of NO_2_^-^ in the oral cavity . . .”**

- Keywords should not duplicate words used in the title. Please revise.

**Keywords have been revised.**

Methods:

- Inclusion of participants during the COVID 19 pandemic would necessitate record of if they had previously been exposed and potientially controlling for any differences. Long covid impacts are still being tested but some studies suggest initial NO altering effects [PMID: 37657532]. Please add this as a limitation of your study.

**Data collection for out study took place in two phases, with n=6 participants studied between November 1st 2019 and March 13th 2020 and n=24 between November 5th 2020 and March 20th 2023. All participants studied during the second phase were required to submit to weekly COVID-19 mitigation testing and then later to be vaccinated as a condition of continued university enrollment. None of the participants experienced a COVID-19 infection during the study, none reported that they were suffering from long COVID, and none reported any reason why they should not perform physical activity (PAR-Q question 7). This information has been added to the manuscript. Nonetheless, we have added the possible impact of COVID-19 as a limitation to the Discussion.**

Table 1: Please add Race and Age data. Minorites reports higher exposure to societal stressors that could impact endogenous NO bioavailability. Also, baseline fitness level and body composition were not considered. Given the limitation of BMI and small sample size, differences in aerobic capacity, lean mass, and adipose could impact NO responses. If data is not available, please add as a limitation.

**Racial and ethnic characteristics and mean age of the participants have been added to the Methods section. Lack of detailed information on aerobic fitness and body composition have been added to the Discussion as an additional limitation.**

Statistical analyses - a sensitivity analysis should be conducted on data provided in figure D. The reader should know if removal of that one individual in the Cetylpyridinium group with the highest pre value changes the statistical significance of the result.

**To determine whether our findings were being driven by the results for that single individual, we further analyzed the data two ways:**

1) **A two-tailed Grubb’s test at P=0.05 was used to test the data for outliers. None were found.**
2) **The two-way ANOVA was repeated excluding that individual’s results. The decrease in % nitrite in the cetylpyridinium group (i.e., from 22±11% to 17±11%) was still significant (i.e., P=0.0071).**

**The above information has been added to the manuscript.**

Secondly, the effect size cited should be labeled (i.e., is it Cohen's d, partial eta, etc.) and the reader should know why it was selected.

**The effect size of 0.15 is actually Cohen’s *f* statistic, which is the appropriate choice for ANOVA. This is now stated in the manuscript and Cohen’s classic textbook cited. To avoid confusing readers by “mixing and matching” different effect size statistics, we have removed reference to the effect size (Hedge’s *g*) from our meta-analysis.**

Results: The focus on percent change is a bit misleading when the individual data points seem to show that one data point is driving the huge change. Please accompany this with the sensitivity analysis data.

**Please see above.**

Discussion: Given the limitations the findings should be toned down. Please remove any mention of these results being generalizable to older adults or patient populations. Further, exercise performance was not tested. A knee extension is more of a functional outcome. Please use contractility as that is what was tested. Lastly, please add the limitation that one time point was tested (static measure) or provide data that salivary NO or muscle function is not under the influence of circadian biology. IF it is under the influence of circadian biology than it is necessary that the reader is made aware that any significant percentage change is outside the normal within or between day fluctuation. Given that salivary cortisol does fluctuate that does bring some concern as to how to interpret a static salivary measure.

**Per the reviewer’s suggestions, we have removed any reference to the generalizability of our results from the Discussion, and refer to muscle contractility only as *a* determinant of exercise performance and/or physical function. We have also added references re. circadian changes (or lack thereof) in our outcome measures, and added the fact that we only tested at one time point as a limitation.**

Reviewer #2: This study investigated the effect of antibacterial mouthwash on muscle contractile function in heathy young adults. The manuscript is well written whilst reviewer has some questions.

**We thank the reviewer for their compliments re. our writing.**

1) What is the significance of examining this study in young adults? In previous studies, the effects of NO3- supplementation on muscle function have been observed in elderly people with impaired endothelial function and people with vascular disease.

**Please see response to reviewer 1.**

2) It is questionable whether the methods used in this experiment were appropriate. In this study, no increase in NO activity was obtained with the use of mouthwash. This study is considered to be an inappropriate experimental design, as it would only be valid if the use of mouthwash increases NO activity.

**The reviewer appears to be confused. As stated in the original manuscript, the purpose of our study was to determine whether over-the-counter mouthwash would *reduce* (not *increase*, as twice suggested by the reviewer above) NO bioavailability, and if so, whether this had any impact on muscle contractile function. Obviously inhibiting the enterosalivary pathway of NO production via mouthwash use would not be expected to increase NO levels.**

3) How reliable is the measurement of breath NO level for assessing NO activity?

**Breath NO levels are a well-established and sensitive indicator of changes in whole-body NO production. A reference has been added to this effect.**

4) Mouthwash was used twice a day, but at what time of the day was it used? Also, was the timing of use standardized for all subjects?

**Participants were instructed to follow the mouthwash manufacturers’ recommendations, which were to use the product morning and evening. This detail has been added to the revised manuscript. Aside from verifying AM and PM use, we did not record the exact timing. As originally stated, however, the effects of cetylpyridinium persist for at least 6 h, which would seem to make the exact timing relatively unimportant.**
