# Supplementary material for: Pilot randomized trial of the effect of antibacterial mouthwash on muscle contractile function in healthy young adults
Source: PLoS One. 2025 Feb 12;20(2):e0312961. doi: 10.1371/journal.pone.0312961 (PMC11819566; doi:10.1371/journal.pone.0312961)
Supplement: Attachment — Submitted filename: Response_to_Reviewers_auresp_2.docx [file pone.0312961.s005.docx]

Reviewer #1: This reviewer is appreciative of the author's responses and believe this manuscript less confusing in its current state. There are only 2 new issues with the manuscript that should be address prior to publication. Well done.

1) As it currently stands, although the overall values are representative of the local population, it is unclear how the 4 minorities are dispersed across the experimental groups. Please add racial/ethnic data and group sample size to table 1 and perform a statistical group comparison and add appropriate p-values to table 1.

**Table 1 has been expanded to provide the information and statistical comparisons requested.**

2) This study does not provide conclusive data to make this claim in line the final paragraph of the manuscript: "Healthy young Athletes seemingly need not be concerned about possible impairments in muscle contractility due to use of cetylpyridinium-based mouthwash.". Please remove or add terms of uncertainty.

**The offending sentence has been replaced with “Short-term use of cetylpyridinium-based mouthwash does not seem to impair whole-body NO bioavailability or muscle contractility in healthy young Individuals.” Note that this is essentially just a restatement of the results.**

Reviewer #3: This is a quite an interesting study. However, the manuscript could benefit from further improvements based on the following comments:

Page 4: Apart from the exclusion criteria, inclusion criteria is to be provided.

**As succinctly stated in the original manuscript, “All other persons were included in the study.” We believe that this already makes it clear that anyone between 18 and 30 y of age, who was not currently using mouthwash, antibiotics, or tobacco products, who had a resting blood pressure > 140/90, who answered no to all of the questions of the Physical Activity Readiness Questionnaire (PAR-Q), and was able to provide informed consent was eligible for inclusion. No changes have therefore been made.**

Page 4: The study design name is to be clearly stated.

**That a parallel-arm design was used is now explicitly stated in the last sentence of the Introduction.**

Page 5 Paragraph 1 Line 4: The name abbreviation could be attached to the 'individual' as a form of identification.

**The initials of the individual responsible for generating the treatment assignment have been added.**

Pahe 5: The baseline, intervention and post time period is to be clearly stated.

**It is unclear what the reviewer expects, as this was an acute study, with the intervention period (i.e., 7 d) already clearly stated. Nonetheless, in response we have added the word “immediately” to indicate that there was no delay between the baseline testing and treatment period, or between the treatment period and post-testing.**

Page 5 Paragraph 1 Line 2: The cut off/requirement or level of acceptance PAR-Q and isokinetic dynamometer is to be stated. The university name is to be stated.

**The name of the university was already explicitly stated just two sentences above, but in response to the reviewer’s comment has also been added here. However, it is completely unclear what they mean by “the cut off/requirement or level of acceptance PAR-Q and isokinetic dynamometer “, so no other changes have been made.**

Page 5 Table 1: The table presentation to follow journal format. The decimal point is to be standardized. Title is too short.

**We have read carefully the guidelines for table formatting (**[**https://journals.plos.org/plosone/s/tables**](https://journals.plos.org/plosone/s/tables)**) and believe that our tables are in conformance. No changes have therefore been made.**

Page 7: ‘the latter after log normalization’ is to be rephrased.

**This sentence has been revised to read “Salivary NO_3_^-^, NO_2_^-^, and log-normalized cGMP concentrations . . .”**

Page 7 Paragraph 2 Line 4 & 6: two-way analysis of variance (ANOVA) and three-way analysis of variance (ANOVA) are to be revised to two-way repeated measure analysis of variance (ANOVA) and three-way repeated measure analysis of variance (ANOVA) respectively. The detail analysis in table form is to be presented e.g. main effects, interactions, effect size, post hoc comparison etc and attached as supplementary table. The statistical test assumptions fulfilment is to be stated.

**Please see below.**

Page 7, Paragraph 2, Line 7: The sentence ‘with treatment as a between-subject factor and velocity and time as within-subject factors’ can be revised to ‘with treatment as a between-subject factor while velocity and time are treated as within-subject factors.

**Both of these comments actually refer to the same two sentences describing the statistical analysis of the data. We believe the fact that repeated measures ANOVAs were performed was already clearly apparent from the nature of the study design and the fact that velocity and time were treated as within-subject factors. Nonetheless, in response to the reviewer’s comment we have now added the words “repeated measures” to both sentences. Full results of these analyses are also now included as a supplement.**

Page 7 Paragraph 2 Line 8: One or two-tailed test for p value is to be stated.

**The use of two-tailed tests is now specified.**

Page 7: The statement ‘According to Cohen [32], this would reflect a “moderate” effect size’ unclear and requires further elaboration. Based on Cohen’s guidelines, Cohen f = 0.15 is considered small and 0.25 considered moderate effect size. 0.15 -0.25 considered small to moderate effect size.

**According to Cohen (pages 285-286 of Ref. 32), f = 0.10 (not 0.15) is considered a “small” effect size. The sentence in question has therefore been revised to read “According to Cohen [32], this would be between a “small” (f = 0.10) and a “medium” (f = 0.25) effect size.”**

Page 7, Paragraph 2: Missing data (if any) is to be reported. If there is no missing data, a statement indicating this is to be included.

**This has been addressed in the following paragraph.**

Page 8 Paragraph 2 Line 5: Indicate clearly the p value in Figure 2D.

**The P value is clearly shown at the top right of panel D, as well as stated in the text.**

Page 8 Paragraph 3 Line 1-3 Muscle contractile function: The results (e.g. p values) are to be clearly presented in the table. For the statement ‘nor were there any significant interaction effects (Table 2)’, the results are to be presented.

**Please see below.**

Table 2: for the table footnote ‘No significant treatment effects were observed’, the p value (s) is/are to be stated/presented. Statistical test is to be denoted in the table footnote. More detailed information such as mean difference, effect size index, p values, 95%CI etc could be presented.

**In response to the reviewer’s comments, P values for isometric/isokinetic torque have been moved from the text to the table, and interaction as well as main effects provided. These changes have made the footnote redundant, so it has been deleted. Conversely, main and interactions effects for Vmax and Pmax have been added to the text for completeness.**
