# Supplementary material for: Pilot randomized trial of the effect of antibacterial mouthwash on muscle contractile function in healthy young adults
Source: PLoS One. 2025 Feb 12;20(2):e0312961. doi: 10.1371/journal.pone.0312961 (PMC11819566; doi:10.1371/journal.pone.0312961)
Supplement: Attachment — Submitted filename: Response_to_Reviewers_auresp_3.docx [file pone.0312961.s006.docx]

Reviewer #3: Minor comment

For Table 1, based on CONSORT statement, statistical tests to compare baseline characteristics between intervention groups are not recommended. Instead, the CONSORT guidelines recommend presenting the baseline data descriptively. As such statistical tests/p values are to be omitted.

**Statistical comparisons between groups have been removed.**

Reviewer #4: The authors in the study examined the effect of antibacterial mouthwash on muscle contractile function in healthy young adults. This study is well done and the manuscript has been revised based on reviewer comments.
I have only two minor comments

P.6. An examination of saliva samples. Typically, during ELISA analysis, duplicate sample analysis is employed for data evaluation. It is essential to incorporate these data regarding the coefficient of variations.

**The CV for these triplicate determinations has now been stated.**

Results
You should add about F value in results of analysis of ANOVA.

**Added.**
