# Supplementary material for: Pilot randomized trial of the effect of antibacterial mouthwash on muscle contractile function in healthy young adults
Source: PLoS One. 2025 Feb 12;20(2):e0312961. doi: 10.1371/journal.pone.0312961 (PMC11819566; doi:10.1371/journal.pone.0312961)
Supplement: Attachment — Submitted filename: Response_to_Reviewers_auresp_4.docx [file pone.0312961.s007.docx]

Reviewer #3: Minor comment

Table 1 footnote: The statement 'P values calculated using Fisher’s exact test and two-tailed unpaired t tests, as appropriate.' is to be omitted.

**The leftover footnote has been removed. (Why the reviewer insisted on further holding acceptance/publication of the paper versus simply letting this be handled at the copyediting stage is completely beyond me.)**
